# Supplementary material for: Epigenetic alterations facilitate transcriptional and translational programs in hypoxia
Source: Nat Cell Biol. 2025 Oct 16;27(11):1965–81. doi: 10.1038/s41556-025-01786-8 (PMC12611764; doi:10.1038/s41556-025-01786-8)
Supplement: Supplementary file 1 — Supplementary Fig. 1 outlining FACS gating strategy. [file 41556_2025_1786_MOESM1_ESM.pdf]

nature cell biology

Supplementary information https://doi.org/10.1038/s41556-025-01786-8

Epigenetic alterations facilitate transcriptional and translational programs in hypoxia

In the format provided by the authors and unedited

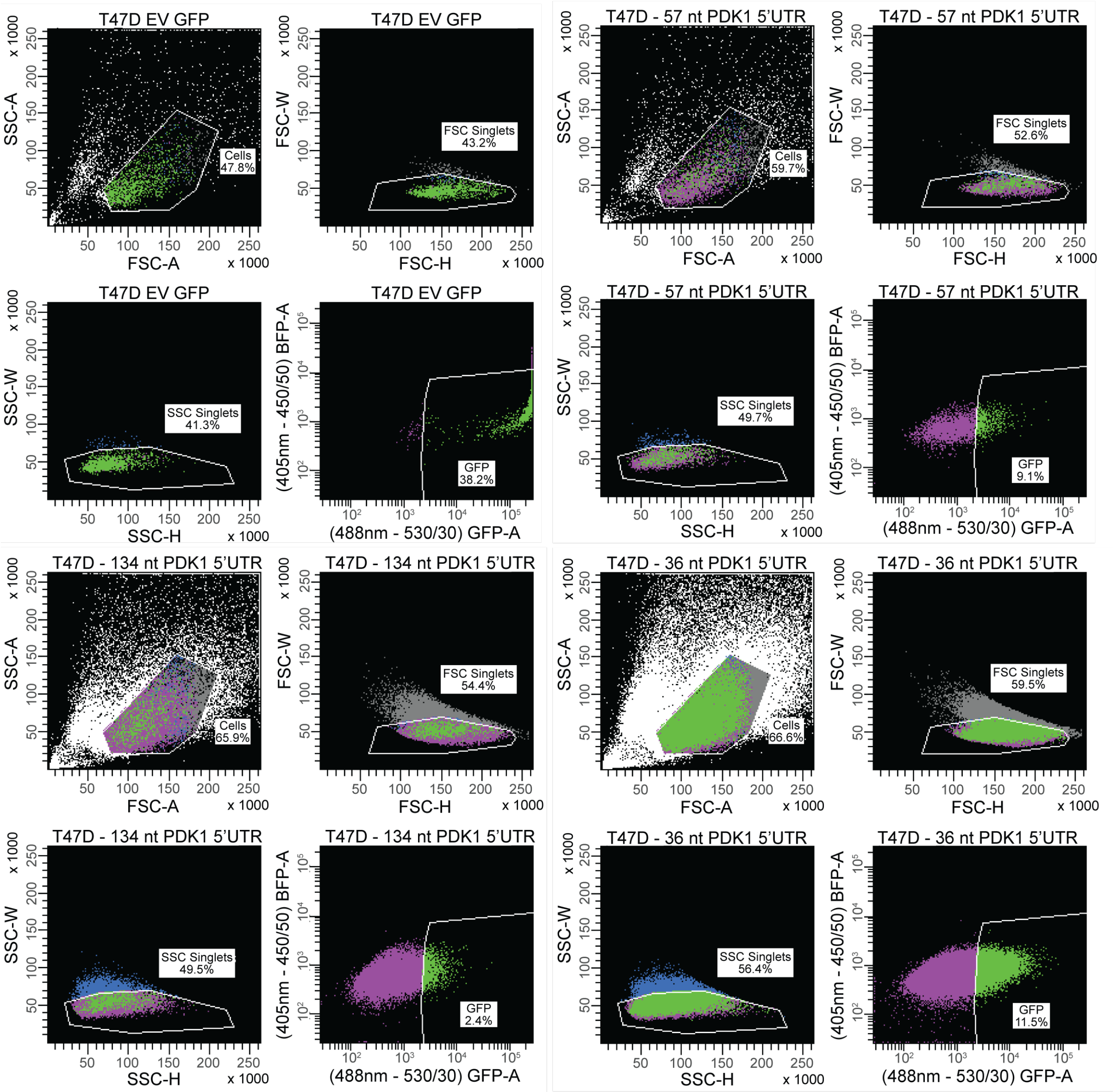

Supplementary Figure 1: Gating strategy for flow cytometry of T47D PDK1 5'UTR CRISPR clones. Successive gating identified FSC and SSC singlets, and finally GFP positive cell populations for T47D cells expressing individual 5'UTR isoforms (36, 57, and 134 nt) of PDK1, and empty vector (EV) controls.
