# Supplementary material for: Epigenetic alterations facilitate transcriptional and translational programs in hypoxia
Source: Nat Cell Biol. 2025 Oct 16;27(11):1965–81. doi: 10.1038/s41556-025-01786-8 (PMC12611764; doi:10.1038/s41556-025-01786-8)
Supplement: Supplementary file 2 — Reporting Summary [file 41556_2025_1786_MOESM2_ESM.pdf]

nature portfolio

Corresponding author(s): Dr. Lynne-Marie Postovit
Dr. Ola Larsson

Last updated by author(s): Aug 3, 2025

Reporting Summary

Nature Portfolio wishes to improve the reproducibility of the work that we publish. This form provides structure for consistency and transparency in reporting. For further information on Nature Portfolio policies, see our Editorial Policies and the Editorial Policy Checklist.

Statistics

For all statistical analyses, confirm that the following items are present in the figure legend, table legend, main text, or Methods section.

n/a	Confirmed	
☐	☒	The exact sample size (n) for each experimental group/condition, given as a discrete number and unit of measurement
☐	☒	A statement on whether measurements were taken from distinct samples or whether the same sample was measured repeatedly
☐	☒	The statistical test(s) used AND whether they are one- or two-sided<br>*Only common tests should be described solely by name; describe more complex techniques in the Methods section.*
☐	☒	A description of all covariates tested
☐	☒	A description of any assumptions or corrections, such as tests of normality and adjustment for multiple comparisons
☐	☒	A full description of the statistical parameters including central tendency (e.g. means) or other basic estimates (e.g. regression coefficient) AND variation (e.g. standard deviation) or associated estimates of uncertainty (e.g. confidence intervals)
☐	☒	For null hypothesis testing, the test statistic (e.g. F, t, r) with confidence intervals, effect sizes, degrees of freedom and P value noted<br>*Give P values as exact values whenever suitable.*
☒	☐	For Bayesian analysis, information on the choice of priors and Markov chain Monte Carlo settings
☒	☐	For hierarchical and complex designs, identification of the appropriate level for tests and full reporting of outcomes
☐	☒	Estimates of effect sizes (e.g. Cohen's d, Pearson's r), indicating how they were calculated

Our web collection on statistics for biologists contains articles on many of the points above.

Software and code

Policy information about availability of computer code

Data collection	Western blot data was collected using ImageStudio Acquisition Software (version 5.2.0) from LICOR (https://www.licor.com/bio/image-studio/).<br>NanoCAGE and Smartseq2 data were acquired using HiSeq Control Software (veriosn 2.2.58/RTA 1.18.64), and NovaSeq Control Software (version 1.6.0)<br>Raw proteomics data were converted to mzML using ProteoWizard Software (version 3.0.22137)<br>GC/MS data acquisition used MassHunter (version 10.2)
Data analysis	All preexisting software used in these studies is described in detail in the methods section, including version numbers and relevant citations.<br><br>Preexisting Pipelines and Software:<br>nf-core chipseq pipeline (version 2.0.0)<br>nf-core atacseq pipeline (version 2.1.2)<br>Bowtie (version 1.2.2)<br>BEDtools (version 2.29.1)<br>NucleoATAC (version 0.3.4)<br>IGV (version 2.8.3)<br>DeepTools (version 3.4.3)<br>deepStats (version 0.3.1)<br>bcl2fastq v2.19<br>bcl2fastq v2.20.0.422<br>BBTools v36.59 (http://sourceforge.net/projects/bbmap/)

HISAT2 (version 2.1.0)
Cytoscape (version 3.8.2.)
ClueGO (version 2.5.8)
TagDust (version 2.33)
Cutadapt (version 1.18)
STREME (version 5.4.1)
Mfold (version 3.6)
Perl script for removal of strand invasion artifacts (from Tang et al. PMID: 23180801)
R (version 4.1.1)
Perl (version 5.16.3)
Agilent MassHunter Quantitative Analysis Software (version 10.2)
QuantStudio Design & Analysis software (version 1.5.2)
QuantStudio Real-time PCR software (version 1.6.1)
DIA-NN (version 1.8.1)

R/Bioconductor Packages used in stand-alone analyses (listed in methods):
RSubread (version 2.6.4)
Anota2seq (version 1.14.0)
changepoint (version 2.2.3)

R/Bioconductor packages that are dependencies of custom software (listed on Code Ocean capsule)
Boruta (version 8.0.0)
R.utils (version 2.13.0)
ROCR (version 1.0-11)
WriteXLS (version 6.7.0)
caret (version 7.0-1)
curl (version 4.3.2)
data.table (version 1.17.0)
dplyr (version 1.1.4)
ggplot2 (version 3.5.1)
ggrepel (version 0.9.6)
gplots (version 3.2.0)
gridExtra (version 2.3)
igraph (version 2.1.4)
phia (version 0.2-1)
plotrix (version 3.8-4)
plyr (version 1.8.9)
qvalue (version 1.26.0)
randomForest (version 4.7-12)
reshape2 (version 1.4.4)
seqinr (version 4.2-36)
shades (version 1.4.0)
stringr (version 1.5.1)
vioplot (version 0.5.1)
grid (version 4.1.1)
rlist (version 0.4.6.2)
matrixStats (version 0.62.0)
RColorBrewer (version 1.1-3)
Hmisc (version 4.7-0)
DESeq2 (version 1.38.3)
RSamtools (version 2.8.0)
GenomicRanges (version 1.44.0)
IRanges (version 2.26.0)
edgR (version 3.34.1)
biomaRt (version 2.48.3)
memes (version 1.0.4)
pqsfinder (version 2.8.0)
limma (version 3.48.3)

Analysis of TSS switching, 5'UTR features, and translatome modelling was carried out using original code in the form of two R software packages. These original R packages, along with data and scripts to reproduce the analysis are available on Code Ocean: https://doi.org/10.24433/CO.4525673.v1

For manuscripts utilizing custom algorithms or software that are central to the research but not yet described in published literature, software must be made available to editors and reviewers. We strongly encourage code deposition in a community repository (e.g. GitHub). See the Nature Portfolio guidelines for submitting code & software for further information.

Data

Policy information about availability of data

All manuscripts must include a data availability statement. This statement should provide the following information, where applicable:

- Accession codes, unique identifiers, or web links for publicly available datasets
- A description of any restrictions on data availability
- For clinical datasets or third party data, please ensure that the statement adheres to our policy

Raw and processed RNA-seq, ChIP-seq, ATAC-seq, and nanoCAGE data have been deposited in the NCBI GEO database under accession number GSE243418. All sequencing datasets were aligned to the NCBI RefSeq GRCh38/hg38 genome assembly, using corresponding RefSeq transcript annotations (release 109, 2020-11-20; https://ftp.ncbi.nlm.nih.gov/refseq/H_sapiens/annotation/annotation_releases/109.20201120/GCF_000001405.39_GRCh38.p13/). Proteomics data have been deposited in the PRIDE database under accession number PXD058655. Metabolite data have been deposited in the MetaboLights repository under accession number MTBLS12086. Other published datasets used in this study can be accessed at GSE11011 (eIF4G-dependent translation signature), GSE115142 (DAP5-dependent translation signature), and GSE76766 (mTOR-dependent translation signature). All other experimental data is provided in the associated Supplementary Data and Source Data files.

Research involving human participants, their data, or biological material

Policy information about studies with human participants or human data. See also policy information about sex, gender (identity/presentation), and sexual orientation and race, ethnicity and racism.

Reporting on sex and gender	N/A
Reporting on race, ethnicity, or other socially relevant groupings	N/A
Population characteristics	N/A
Recruitment	N/A
Ethics oversight	N/A

Note that full information on the approval of the study protocol must also be provided in the manuscript.

Field-specific reporting

Please select the one below that is the best fit for your research. If you are not sure, read the appropriate sections before making your selection.

☒ Life sciences ☐ Behavioural & social sciences ☐ Ecological, evolutionary & environmental sciences

For a reference copy of the document with all sections, see nature.com/documents/nr-reporting-summary-flat.pdf

Life sciences study design

All studies must disclose on these points even when the disclosure is negative.

Sample size	No statistical methods were used to pre-determine sample sizes, but sample sizes are comparable to those previously published (PMID 32427827) and standard in the field. All analyses were performed on data from 3 or more independent experiments, with the exception of isoform-selective RT-qPCR data presented in Figure 7f, and protein/mRNA quantifications for CRISPR clones presented in Figure 7j, where 2 independent experiments were performed. In these instances, an ANOVA was used to identify if expression/translation of all isoforms is the same, or different. As such, the residual degrees of freedom in the models for data in Figures 7f and j were 6, and 16, respectively, which provide sufficient statistical power for this approach to be valid. Furthermore, for data presented in Figure 7j, multiple CRISPR clones were used for each 5'UTR isoform. Therefore, in addition to independent experimental replicates, we also have included biological replicates.
Data exclusions	Data from one sample of nanoCAGE sequencing in T47D cells under normoxia was excluded due to sequencing failure. This was determined by a lack of sequencing reads after data processing and alignment. Data from one replicate of nanoCAGE sequencing in H9 cells under hypoxia and normoxia was excluded due to being an outlier upon PCA.
Replication	All attempts to replicate data were successful. The number of repeats is described in the figure legends. For sequencing-based data, the reproducibility of replicates was assessed using Principle Component Analysis, hierarchical clustering, and correlation analyses between samples.
Randomization	No randomization was performed in this study. Covariates were controlled for in differential expression analyses by including batch/replicate in linear regression models (detailed in the methods).
Blinding	Blinding was not relevant to the study design as none of the analyses involved any type of scoring that could be subjective, or vary between individuals carrying out the analysis.

Reporting for specific materials, systems and methods

We require information from authors about some types of materials, experimental systems and methods used in many studies. Here, indicate whether each material, system or method listed is relevant to your study. If you are not sure if a list item applies to your research, read the appropriate section before selecting a response.

Materials & experimental systems

n/a	Involved in the study
☐	☒ Antibodies
☐	☒ Eukaryotic cell lines
☒	☐ Palaeontology and archaeology
☒	☐ Animals and other organisms
☒	☐ Clinical data
☒	☐ Dual use research of concern
☒	☐ Plants

Methods

n/a	Involved in the study
☐	☒ ChIP-seq
☐	☒ Flow cytometry
☒	☐ MRI-based neuroimaging

Antibodies

Antibodies used

4E-BP1, phospho-S65 (Cell signaling; Cat# 9451S; 1:1000)
4E-BP1 (35H11) (Cell signaling; Cat# 9644; 1:7000)
S6 ribosomal protein, phospho-S240/244 (Cell signaling; Cat#2215; 1:1000)
S6 ribosomal protein (5G10) (Cell signaling; Cat# 2217; 1:1000)
EIF2S1 [E90] phospho-S51 (Abcam; Cat# ab32157; 1:1000)
eIF2alpha (Cell signaling; Cat# 9722; 1:1000)
HIF1alpha (D1S7W) XP (Cell signaling; Cat# 36169; 1:1000)
PDK1 [4A11] (Abcam; Cat# ab110025; 1:1000)
PGK1 (Abcam; Cat# ab38007; 1:1000
PDHE1alpha (D6) (Santa Cruz; Cat# sc-277092; 1:1000)
PDHE1alpha phospho-S232 (EMD milipore; Cat# AP1063-50ug; 1:1000)
PDHE1alpha phospho-S293 [EPR12200] (Abcam; Cat# ab177461; 1:1000)
Histone H3 (D1H2) XP (Cell signaling; Cat# 4499S; 1:1000)
Histone H3K4me3 (C42D8) (Cell signaling; Cat# 9751S; 1:1000)
H3K4me3 (ChIPseq) (Epigentek; Cat #A4033; 6 ug per ChIP sample)
Alpha-tubulin (mouse) (LICOR; Cat# 926-42213; 1:4000)
Beta-tubulin (rabbit) (LICOR; Cat# 926-42211; 1:4000)
Beta-actin (mouse) (LICOR; Cat# 926-42212; 1:4000)
Beta-actin (rabbit) (LICOR; Cat# 926-42210; 1:4000)
IRDye 800CW anti-mouse (LICOR; Cat# 926-32212; 1:10000)
IRDye 680RD anti-mouse (LICOR; Cat# 926-68072; 1:10000)
IRDye 800CW anti-rabbit (LICOR; Cat# 926-32213; 1:10000)
IRDye 680RD anti-rabbit (LICOR; Cat# 926-68073; 1:10000)

Validation

4E-BP1, phospho-S65 Cell signaling Cat# 9451S, 1:1000, Western blot analysis of extracts from 293 cells that were starved for 24 hours in serum-free medium and underwent a 1-hour amino acid deprivation. Amino acids were replenished for 1 hour. Cells were then either untreated or treated with 100 nM insulin for 30 minutes.

4E-BP1 (35H11) Cell signaling Cat# 9644, 1:7000, Western blot analysis of extracts from control HeLa cells or HeLa cells with a targeted mutation in the gene encoding 4E-BP1.

S6 ribosomal protein, phospho-S240/244 Cell signaling Cat#2215, 1:1000, Western blot analysis of extracts from 293 cells, untreated or treated with 20% FBS for 10, 20 or 30 min.

S6 ribosomal protein (5G10) Cell signaling Cat# 2217, 1:1000, Western blot analysis of extracts from HeLa, NIH/3T3, PC12 and COS cells.

EIF2S1 [E90] phospho-S51 Abcam Cat# ab32157, 1:1000, Western blot of whole cell lysates of RAW 264.7 treated with 5 ug/ml tunicamycin for 18 hours (positive control) or treated with 5 ug/ml tunicamycin for 18 hours and treated with alkaline phosphatase or. Lambda phosphatase (negative control).

eIF2alpha Cell signaling Cat# 9722, 1:1000, Western blot analysis of extracts from PC12 cells.

HIF1alpha (D1S7W) XP Cell signaling Cat# 36169, 1:1000, Western blot analysis of extracts from Hep G2 cells untreated or treated with cobalt chloride (100 μM, 4 h; +), Raji cells untreated or treated with cobalt chloride (100 μM, 4 h; +) and U-2 OS cells untreated or treated with DMOG (1 mM, 6 h; +)

PDK1 [4A11] Abcam Cat# ab110025, 1:1000, Western blot analysis of extracts from NIH/3T3, HeLa, Jurkat, HepG2,PC-12, COS7 cells.

PGK1 Abcam Cat# ab38007, 1:1000, discontinued, Western blot analysis of extracts from mouse stomach tissue, HepG2 cells.

PDHE1alpha (D6) Santa Cruz Cat# sc-277092,1:1000, Western blot analysis of PDH-E1α expression in Hep G2, HeLa, Sol8, C2C12, L8, L6, EOC 20, H4, IMR-3 and Hep G2 whole cell lysates.

PDHE1alpha phospho-S232 EMD milipore Cat# AP1063-50ug, 1:1000, Whole tissue extract from mouse liver (20 µg) left untreated or treated with dichloroacetate (DCA, 5 mM for 4 h).

PDHE1alpha phospho-S293 [EPR12200] Abcam Cat# ab177461, 1:1000, Western blot analysis of extracts from rat kidneys untreated or treated with phosphatase, or HT-29 cells treated with 8 mM Sodium butyrate for 24 hours without or with phosphatase treatment.

Histone H3 (D1H2) XP Cell signaling Cat# 4499S, 1:1000, Western blot analysis of extracts from HeLa, NIH/3T3, C6, COS cells.

Histone H3K4me3 (C42D8, for WB) Cell signaling Cat# 9751S, 1:1000, HeLa and NIH/3T3 cell lysates were probed with Tri-Methyl Histone H3 (Lys4) (C42D8) Rabbit mAb or Tri-Methyl Histone H3 (Lys4) Rabbit mAb pre-adsorbed with 1.5 µM of various competitor peptides, only the tri-methyl histone H3 (Lys4) peptide competed away binding of the antibody.

H3K4me3 (ChIPseq) Epigentek Cat #A4033, 6µg per ChIP sample, Chromatin immunoprecipitation analysis of extracts of 293F cells was performed using Histone H3K4me3 (H3K4 Trimethyl) Polyclonal Antibody. The amount of immunoprecipitated DNA was checked by quantitative PCR. Additional validation was performed in PMID: 30244833.

Eukaryotic cell lines

Policy information about cell lines and Sex and Gender in Research

Cell line source(s)	T47D ductal carcinoma cells, female; ATCC (HTB-133)<br>HEK 293T cells, female; ATCC (CRL-3216)<br>H9 human embryonic stem cells, female; WiCell (WA09)
Authentication	All cell lines were authenticated by STR profiling at the SickKids Research Institute (Toronto, Ontario, Canada).
Mycoplasma contamination	All cells routinely tested negative for mycoplasma (Mycoplasma detection kit, ATCC).
Commonly misidentified lines<br>(See ICLAC register)	No commonly misidentified cell lines were used in this study.

Plants

Seed stocks	N/A
Novel plant genotypes	N/A
Authentication	N/A

ChIP-seq

Data deposition

☒ Confirm that both raw and final processed data have been deposited in a public database such as GEO.

☒ Confirm that you have deposited or provided access to graph files (e.g. BED files) for the called peaks.

Data access links<br>*May remain private before publication.*	All raw and processed ChIP-seq data generated in this study is available on the GEO database with accession GSE243418.
Files in database submission	Raw sequencing data:<br>H9_H_H3K4me3_1_R1.fastq.gz<br>H9_H_H3K4me3_1_R2.fastq.gz<br>H9_H_H3K4me3_2_R1.fastq.gz<br>H9_H_H3K4me3_2_R2.fastq.gz<br>H9_H_H3K4me3_3_R1.fastq.gz<br>H9_H_H3K4me3_3_R2.fastq.gz<br>H9_H_input_1_R1.fastq.gz<br>H9_H_input_1_R2.fastq.gz<br>H9_H_input_2_R1.fastq.gz<br>H9_H_input_2_R2.fastq.gz

H9_H_input_3_R1.fastq.gz
H9_H_input_3_R2.fastq.gz
H9_N_H3K4me3_1_R1.fastq.gz
H9_N_H3K4me3_1_R2.fastq.gz
H9_N_H3K4me3_2_R1.fastq.gz
H9_N_H3K4me3_2_R2.fastq.gz
H9_N_H3K4me3_3_R1.fastq.gz
H9_N_H3K4me3_3_R2.fastq.gz
H9_N_input_1_R1.fastq.gz
H9_N_input_1_R2.fastq.gz
H9_N_input_2_R1.fastq.gz
H9_N_input_2_R2.fastq.gz
H9_N_input_3_R1.fastq.gz
H9_N_input_3_R2.fastq.gz
T47D_H_H3K4me3_1_R1.fastq.gz
T47D_H_H3K4me3_1_R2.fastq.gz
T47D_H_H3K4me3_2_R1.fastq.gz
T47D_H_H3K4me3_2_R2.fastq.gz
T47D_H_H3K4me3_3_R1.fastq.gz
T47D_H_H3K4me3_3_R2.fastq.gz
T47D_H_input_1_R1.fastq.gz
T47D_H_input_1_R2.fastq.gz
T47D_H_input_2_R1.fastq.gz
T47D_H_input_2_R2.fastq.gz
T47D_H_input_3_R1.fastq.gz
T47D_H_input_3_R2.fastq.gz
T47D_N_H3K4me3_1_R1.fastq.gz
T47D_N_H3K4me3_1_R2.fastq.gz
T47D_N_H3K4me3_2_R1.fastq.gz
T47D_N_H3K4me3_2_R2.fastq.gz
T47D_N_H3K4me3_3_R1.fastq.gz
T47D_N_H3K4me3_3_R2.fastq.gz
T47D_N_input_1_R1.fastq.gz
T47D_N_input_1_R2.fastq.gz
T47D_N_input_2_R1.fastq.gz
T47D_N_input_2_R2.fastq.gz
T47D_N_input_3_R1.fastq.gz
T47D_N_input_3_R2.fastq.gz
NS.X0096.004.IDT_i7_34---IDT_i5_34.DSP1951_05_24h_IP_rep1_R1.fastq.gz
NS.X0096.004.IDT_i7_34---IDT_i5_34.DSP1951_05_24h_IP_rep1_R2.fastq.gz
NS.X0096.004.IDT_i7_40---IDT_i5_40.DSP1951_11_24h_IP_rep2_R1.fastq.gz
NS.X0096.004.IDT_i7_40---IDT_i5_40.DSP1951_11_24h_IP_rep2_R2.fastq.gz
NS.X0096.004.IDT_i7_46---IDT_i5_46.DSP1951_17_24h_IP_rep3_R1.fastq.gz
NS.X0096.004.IDT_i7_46---IDT_i5_46.DSP1951_17_24h_IP_rep3_R2.fastq.gz
NS.X0096.004.IDT_i7_52---IDT_i5_52.DSP1951_23_24h_IP_rep4_R1.fastq.gz
NS.X0096.004.IDT_i7_52---IDT_i5_52.DSP1951_23_24h_IP_rep4_R2.fastq.gz
NS.X0096.004.IDT_i7_31---IDT_i5_31.DSP1951_02_24h-_input_rep1_R1.fastq.gz
NS.X0096.004.IDT_i7_31---IDT_i5_31.DSP1951_02_24h-_input_rep1_R2.fastq.gz
NS.X0096.004.IDT_i7_37---IDT_i5_37.DSP1951_08_24h_input_rep2_R1.fastq.gz
NS.X0096.004.IDT_i7_37---IDT_i5_37.DSP1951_08_24h_input_rep2_R2.fastq.gz
NS.X0096.004.IDT_i7_43---IDT_i5_43.DSP1951_14_24h_input_rep3_R1.fastq.gz
NS.X0096.004.IDT_i7_43---IDT_i5_43.DSP1951_14_24h_input_rep3_R2.fastq.gz
NS.X0096.004.IDT_i7_49---IDT_i5_49.DSP1951_20_24h_input_rep4_R1.fastq.gz
NS.X0096.004.IDT_i7_49---IDT_i5_49.DSP1951_20_24h_input_rep4_R2.fastq.gz
NS.X0096.004.IDT_i7_33---IDT_i5_33.DSP1951_04_0h_IP_rep1_R1.fastq.gz
NS.X0096.004.IDT_i7_33---IDT_i5_33.DSP1951_04_0h_IP_rep1_R2.fastq.gz
NS.X0096.004.IDT_i7_39---IDT_i5_39.DSP1951_10_0h_IP_rep2_R1.fastq.gz
NS.X0096.004.IDT_i7_39---IDT_i5_39.DSP1951_10_0h_IP_rep2_R2.fastq.gz
NS.X0096.004.IDT_i7_45---IDT_i5_45.DSP1951_16_0h_IP_rep3_R1.fastq.gz
NS.X0096.004.IDT_i7_45---IDT_i5_45.DSP1951_16_0h_IP_rep3_R2.fastq.gz
NS.X0096.004.IDT_i7_51---IDT_i5_51.DSP1951_22_0h_IP_rep4_R1.fastq.gz
NS.X0096.004.IDT_i7_51---IDT_i5_51.DSP1951_22_0h_IP_rep4_R2.fastq.gz
NS.X0096.004.IDT_i7_30---IDT_i5_30.DSP1951_01_0h_input_rep1_R1.fastq.gz
NS.X0096.004.IDT_i7_30---IDT_i5_30.DSP1951_01_0h_input_rep1_R2.fastq.gz
NS.X0096.004.IDT_i7_36---IDT_i5_36.DSP1951_07_0h_input_rep2_R1.fastq.gz
NS.X0096.004.IDT_i7_36---IDT_i5_36.DSP1951_07_0h_input_rep2_R2.fastq.gz
NS.X0096.004.IDT_i7_42---IDT_i5_42.DSP1951_13_0h_input_rep3_R1.fastq.gz
NS.X0096.004.IDT_i7_42---IDT_i5_42.DSP1951_13_0h_input_rep3_R2.fastq.gz
NS.X0096.004.IDT_i7_48---IDT_i5_48.DSP1951_19_0h_input_rep4_R1.fastq.gz
NS.X0096.004.IDT_i7_48---IDT_i5_48.DSP1951_19_0h_input_rep4_R2.fastq.gz

Processed data, aligned to human 5'UTR regions:
H9_H_H3K4me3_R1.mLb.clN.sorted.trimmed95.cdf
H9_H_H3K4me3_R2.mLb.clN.sorted.trimmed95.cdf
H9_H_H3K4me3_R3.mLb.clN.sorted.trimmed95.cdf
H9_H_input_R1.mLb.clN.sorted.trimmed95.cdf

H9_H_input_R2.mLb.clN.sorted.trimmed95.cdf
H9_H_input_R3.mLb.clN.sorted.trimmed95.cdf
H9_N_H3K4me3_R1.mLb.clN.sorted.trimmed95.cdf
H9_N_H3K4me3_R2.mLb.clN.sorted.trimmed95.cdf
H9_N_H3K4me3_R3.mLb.clN.sorted.trimmed95.cdf
H9_N_input_R1.mLb.clN.sorted.trimmed95.cdf
H9_N_input_R2.mLb.clN.sorted.trimmed95.cdf
H9_N_input_R3.mLb.clN.sorted.trimmed95.cdf
T47D_H_H3K4me3_R1.mLb.clN.sorted.trimmed95.cdf
T47D_H_H3K4me3_R2.mLb.clN.sorted.trimmed95.cdf
T47D_H_H3K4me3_R3.mLb.clN.sorted.trimmed95.cdf
T47D_H_input_R1.mLb.clN.sorted.trimmed95.cdf
T47D_H_input_R2.mLb.clN.sorted.trimmed95.cdf
T47D_H_input_R3.mLb.clN.sorted.trimmed95.cdf
T47D_N_H3K4me3_R1.mLb.clN.sorted.trimmed95.cdf
T47D_N_H3K4me3_R2.mLb.clN.sorted.trimmed95.cdf
T47D_N_H3K4me3_R3.mLb.clN.sorted.trimmed95.cdf
T47D_N_input_R1.mLb.clN.sorted.trimmed95.cdf
T47D_N_input_R2.mLb.clN.sorted.trimmed95.cdf
T47D_N_input_R3.mLb.clN.sorted.trimmed95.cdf
C48_24hr_H3K4me3_rep1.mLb.clN.sorted.trimmed95.cumsum.cdf
C48_24hr_H3K4me3_rep2.mLb.clN.sorted.trimmed95.cumsum.cdf
C48_24hr_H3K4me3_rep3.mLb.clN.sorted.trimmed95.cumsum.cdf
C48_24hr_H3K4me3_rep4.mLb.clN.sorted.trimmed95.cumsum.cdf
C48_24hr_input_rep1.mLb.clN.sorted.trimmed95.cumsum.cdf
C48_24hr_input_rep2.mLb.clN.sorted.trimmed95.cumsum.cdf
C48_24hr_input_rep3.mLb.clN.sorted.trimmed95.cumsum.cdf
C48_24hr_input_rep4.mLb.clN.sorted.trimmed95.cumsum.cdf
C48_0hr_H3K4me3_rep1.mLb.clN.sorted.trimmed95.cumsum.cdf
C48_0hr_H3K4me3_rep2.mLb.clN.sorted.trimmed95.cumsum.cdf
C48_0hr_H3K4me3_rep3.mLb.clN.sorted.trimmed95.cumsum.cdf
C48_0hr_H3K4me3_rep4.mLb.clN.sorted.trimmed95.cumsum.cdf
C48_0hr_input_rep1.mLb.clN.sorted.trimmed95.cumsum.cdf
C48_0hr_input_rep2.mLb.clN.sorted.trimmed95.cumsum.cdf
C48_0hr_input_rep3.mLb.clN.sorted.trimmed95.cumsum.cdf
C48_0hr_input_rep4.mLb.clN.sorted.trimmed95.cumsum.cdf

Processed data, called peaks:
H9_H_H3K4me3_rep1_peaks.narrowPeak
H9_H_H3K4me3_rep2_peaks.narrowPeak
H9_H_H3K4me3_rep3_peaks.narrowPeak
H9_N_H3K4me3_rep1_peaks.narrowPeak
H9_N_H3K4me3_rep2_peaks.narrowPeak
H9_N_H3K4me3_rep3_peaks.narrowPeak
T47D_H_H3K4me3_rep1_peaks.narrowPeak
T47D_H_H3K4me3_rep2_peaks.narrowPeak
T47D_H_H3K4me3_rep3_peaks.narrowPeak
T47D_N_H3K4me3_rep1_peaks.narrowPeak
T47D_N_H3K4me3_rep2_peaks.narrowPeak
T47D_N_H3K4me3_rep3_peaks.narrowPeak
C48_24hr_H3K4me3_rep1_peaks.narrowPeak
C48_24hr_H3K4me3_rep2_peaks.narrowPeak
C48_24hr_H3K4me3_rep3_peaks.narrowPeak
C48_24hr_H3K4me3_rep4_peaks.narrowPeak
C48_0hr_H3K4me3_rep1_peaks.narrowPeak
C48_0hr_H3K4me3_rep2_peaks.narrowPeak
C48_0hr_H3K4me3_rep3_peaks.narrowPeak
C48_0hr_H3K4me3_rep4_peaks.narrowPeak

Genome browser session
(e.g. UCSC)

No longer applicable.

Methodology

Replicates

N = 3 for each of two cell lines (T47D and H9) in hypoxia and normoxia. N = 4 for T47D cells treated with either DMSO (0 h) or compound-48 (C48) for 24 h. Reproducibility of the replicates was assessed using Principle Component Analysis (results provided in Extended Data Figure 6), hierarchical clustering, and correlations between samples.

Sequencing depth

Reads are 101bp, with a paired-end setup. Total raw reads are as follows:

H9_H_H3K4me3_R1: 74635732
H9_H_H3K4me3_R2: 80219516
H9_H_H3K4me3_R3: 78220806
H9_H_input_R1: 295713012
H9_H_input_R2: 255300374
H9_H_input_R3: 214645350

H9_N_H3K4me3_R1: 70275862
H9_N_H3K4me3_R2: 70588338
H9_N_H3K4me3_R3: 67492356
H9_N_input_R1: 223681710
H9_N_input_R2: 186776302
H9_N_input_R3: 228174104
T47D_H_H3K4me3_R1: 77663460
T47D_H_H3K4me3_R2: 79771312
T47D_H_H3K4me3_R3: 80211274
T47D_H_input_R1: 277232374
T47D_H_input_R2: 271966508
T47D_H_input_R3: 172351612
T47D_N_H3K4me3_R1: 86659118
T47D_N_H3K4me3_R2: 68534990
T47D_N_H3K4me3_R3: 88714902
T47D_N_input_R1: 274816836
T47D_N_input_R2: 312448620
T47D_N_input_R3: 258789032
C48_0hr_H3K4me3_rep1: 84394309
C48_0hr_H3K4me3_rep2: 79764369
C48_0hr_H3K4me3_rep3: 86199711
C48_0hr_H3K4me3_rep4: 76325166
C48_0hr_input_rep1: 90571962
C48_0hr_input_rep2: 80991338
C48_0hr_input_rep3: 77365219
C48_0hr_input_rep4: 88295985
C48_24hr_H3K4me3_rep1: 95161865
C48_24hr_H3K4me3_rep2: 78654392
C48_24hr_H3K4me3_rep3: 90864553
C48_24hr_H3K4me3_rep4: 81468184
C48_24hr_input_rep1: 89732670
C48_24hr_input_rep2: 94003924
C48_24hr_input_rep3: 88090559
C48_24hr_input_rep4: 88702605
C48_48hr_H3K4me3_rep1: 88191753
C48_48hr_H3K4me3_rep2: 83774451
C48_48hr_H3K4me3_rep3: 82371767
C48_48hr_H3K4me3_rep4: 79573865
C48_48hr_input_rep1: 91941255
C48_48hr_input_rep2: 114668477
C48_48hr_input_rep3: 95640688
C48_48hr_input_rep4: 70614428

Uniquely mapped reads are as follows (following removal of ENCODE blacklist regions):
H9_H_H3K4me3_R1: 37317866
H9_H_H3K4me3_R2: 40109758
H9_H_H3K4me3_R3: 39110403
H9_H_input_R1: 147856506
H9_H_input_R2: 127650187
H9_H_input_R3: 107322675
H9_N_H3K4me3_R1: 35137931
H9_N_H3K4me3_R2: 35294169
H9_N_H3K4me3_R3: 33746178
H9_N_input_R1: 111840855
H9_N_input_R2: 93388151
H9_N_input_R3: 114087052
T47D_H_H3K4me3_R1: 38831730
T47D_H_H3K4me3_R2: 39885656
T47D_H_H3K4me3_R3: 40105637
T47D_H_input_R1: 138616187
T47D_H_input_R2: 135983254
T47D_H_input_R3: 86175806
T47D_N_H3K4me3_R1: 43329559
T47D_N_H3K4me3_R2: 34267495
T47D_N_H3K4me3_R3: 44357451
T47D_N_input_R1: 137408418
T47D_N_input_R2: 156224310
T47D_N_input_R3: 129394516
T47D_N_input_R3_T1 145243816
C48_0hr_H3K4me3_rep1 65610320
C48_0hr_H3K4me3_rep2 62061305
C48_0hr_H3K4me3_rep3 66398742
C48_0hr_H3K4me3_rep4 59306964
C48_0hr_input_rep1 72241012
C48_0hr_input_rep2 64409241

C48_0hr_input_rep3 62040919
C48_0hr_input_rep4 70378628
C48_24hr_H3K4me3_rep1 73383442
C48_24hr_H3K4me3_rep2 62086313
C48_24hr_H3K4me3_rep3 70315745
C48_24hr_H3K4me3_rep4 63418790
C48_24hr_input_rep1 71742298
C48_24hr_input_rep2 75450342
C48_24hr_input_rep3 70456718
C48_24hr_input_rep4 70800600
C48_48hr_H3K4me3_rep1 68261352
C48_48hr_H3K4me3_rep2 65382533
C48_48hr_H3K4me3_rep3 64106221
C48_48hr_H3K4me3_rep4 62782519
C48_48hr_input_rep1 73325561
C48_48hr_input_rep2 91978308
C48_48hr_input_rep3 76865323
C48_48hr_input_rep4 56512534

Antibodies

The antibody used for ChIP-seq was H3K4me3, Epigentek Cat# A4033.

Peak calling parameters

The results presented in the study did not employ traditional peak-calling-based analysis of ChIP-seq data. Our methods are describe in detail in the methods section, and all processed files required to reproduce the analysis are provided with the GEO submission above. However, the quality of the data was assessed by the traditional metrics based on peak-calling with MACS2. To do this, the nf-core ChIP-seq pipeline (version 2.0.0) was used with default settings, using the command:
nextflow run nf-core/chipseq --fasta GCF_000001405.39_GRCh38.p13_genomic.fna --gtf GCF_000001405.39_GRCh38.p13_genomic.gtf --macs_gsize 2805636231 --blacklist hg38-blacklist.v2.refseq.bed ---aligner star --narrow_peak --macs_fdr 0.15 --min_reps_consensus 3 (n replicates - 1)

Data quality

The quality of the data was assessed using fingerprint plots and read distribution profiles after alignment, annotation and filtering.

The number of peaks called by MACS2 passing thresholds are as follows:
T47D_H_H3K4me3_R1: 73993
T47D_H_H3K4me3_R2: 54848
T47D_H_H3K4me3_R3: 57825
T47D_N_H3K4me3_R1: 42231
T47D_N_H3K4me3_R2: 30953
T47D_N_H3K4me3_R3: 38387
H9_H_H3K4me3_R1: 54884
H9_H_H3K4me3_R2: 24479
H9_H_H3K4me3_R3: 23075
H9_N_H3K4me3_R1:64942
H9_N_H3K4me3_R2: 39403
H9_N_H3K4me3_R3: 25606
C48_0hr_H3K4me3_rep1: 112791
C48_0hr_H3K4me3_rep2: 111564
C48_0hr_H3K4me3_rep3: 112058
C48_0hr_H3K4me3_rep4: 117266
C48_24hr_H3K4me3_rep1: 135962
C48_24hr_H3K4me3_rep2: 134304
C48_24hr_H3K4me3_rep3: 131786
C48_24hr_H3K4me3_rep4: 135549

Software

Raw H3K4me3 fastq files were processed using the nf-core/chipseq pipeline v.2.0.0 (available at https://github.com/nf-core/chipseq). Reads were aligned to the NCBI RefSeq GRCh38/hg38 genome assembly (release 109, 2020-11-20; https://ftp.ncbi.nlm.nih.gov/refseq/H_sapiens/annotation/annotation_releases/109.20201120/GCF_000001405.39_GRCh38.p13/) in paired-end mode with the following settings: --macs_gsize 2805636231 --blacklist hg38-blacklist.v2.refseq.bed ---aligner star --narrow_peak --macs_fdr 0.15 --min_reps_consensus 3 (n replicates - 1). The output of the pipeline includes BAM files with aligned reads with duplicates, multi-mapping, unpaired, and ENCODE blacklist reads filtered out. In order to assess shifts in the position of H3K4me3 marks relative to TSSs of protein-coding genes, filtered aligned reads were extracted from the BAM files and re-aligned to a custom index of 5’UTR genomic sequences using Bowtie (version 1.2.2) (settings: -v 2 -X 1000 -a). Bedtools genomeCoverageBed (v2.29.1) was then used to compute the genome coverage of H3K4me3 with -dz and -pc options, and -scale to normalize for library size. The cumulative sum of H3K4me3 coverage was calculated for each genomic region corresponding to RefSeq transcripts, and genomic regions were trimmed at the nucleotide position where 95% of the cumulative H3K4me3 signal had already occurred. All fully process ChIP-seq files used in the analysis are provided at the GEO accession GSE243418.

Flow Cytometry

Plots

Confirm that:

☒ The axis labels state the marker and fluorochrome used (e.g. CD4-FITC).

☒ The axis scales are clearly visible. Include numbers along axes only for bottom left plot of group (a 'group' is an analysis of identical markers).

☒ All plots are contour plots with outliers or pseudocolor plots.

☒ A numerical value for number of cells or percentage (with statistics) is provided.

Methodology

Sample preparation	See Methods section for origin and genetic manipulation of T47D cells. For fluorescence-activated cell sorting (FACS) cells were passaged three times after stable transduction, collected after trypsinization, and resuspended in PBS containing 2% FBS. Subsequently, cells were delivered to Lady Davis Institute (LDI) Flow Cytometry Facility for sorting. Approximately 1 x 104 cells were sorted at 30 psi with a 100 µm nozzle into duplicate wells of a 6-well plate containing 2 mL of growth media.
Instrument	BD FACSAria Fusion cell sorter
Software	FACSDiva Software Version 8.0.2
Cell population abundance	~ 2.0e4
Gating strategy	Gating strategy was determined by the Lady Davis Institute Flow Cytometry Facility, where successive gating identified FSC Singlets, SSC Singlets, and finally GFP positive cells (see explanatory panels in Supplementary Fig. 1 in Supplementary Information).

☒ Tick this box to confirm that a figure exemplifying the gating strategy is provided in the Supplementary Information.
