# Supplementary material for: Epigenetic alterations facilitate transcriptional and translational programs in hypoxia
Source: Nat Cell Biol. 2025 Oct 16;27(11):1965–81. doi: 10.1038/s41556-025-01786-8 (PMC12611764; doi:10.1038/s41556-025-01786-8)

Figure 2 a)

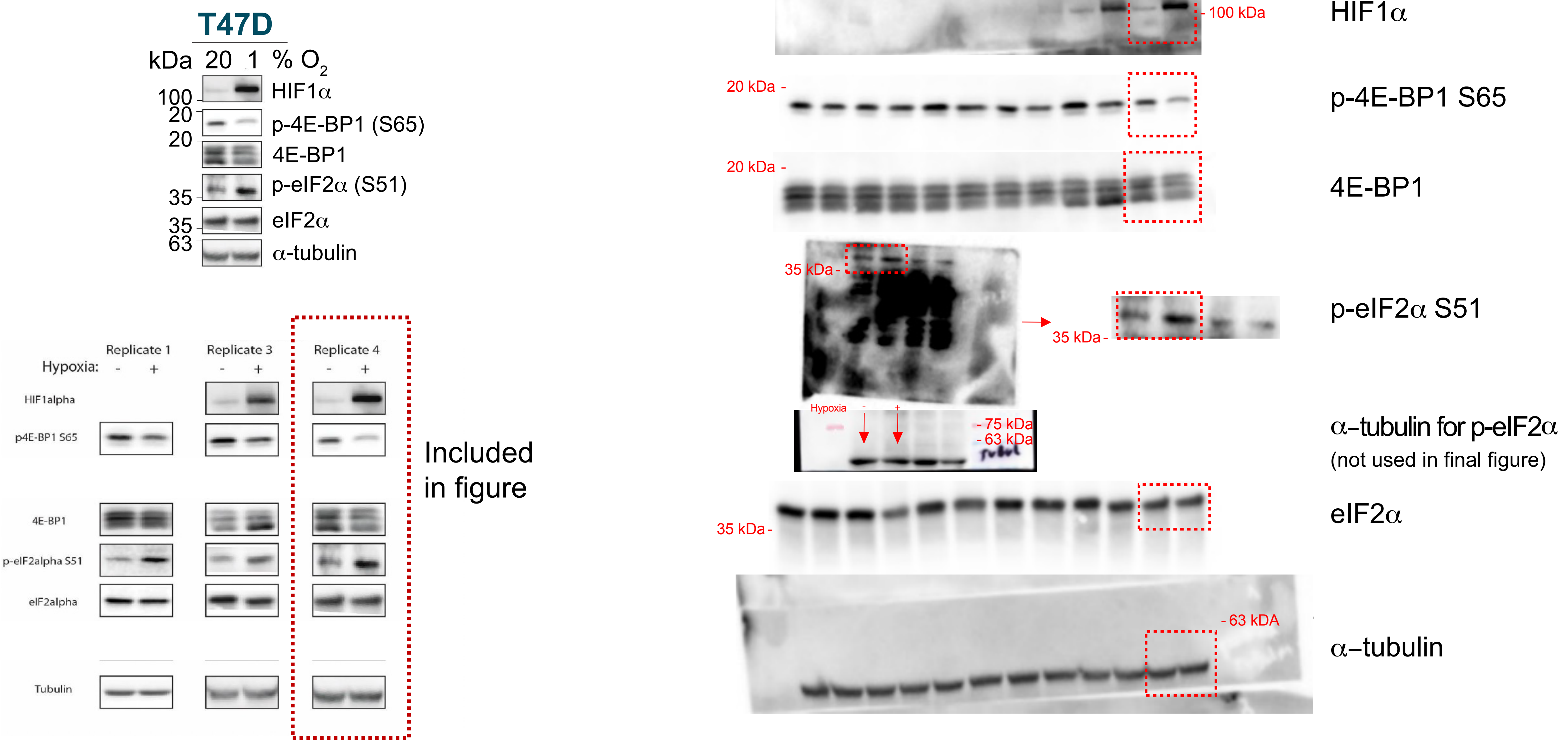
T47D
kDa 20 1 % O2
100
20
20
35
35
63
HIF1α
p-4E-BP1 (S65)
4E-BP1
p-eIF2α (S51)
eIF2α
α-tubulin
Replicate 1
Replicate 3
Replicate 4
Hypoxia: - + - + - +
HIF1alpha
p4E-BP1 S65
4E-BP1
p-eIF2alpha S51
eIF2alpha
Tubulin
Included in figure
Hypoxia - +
100 kDa
HIF1α
20 kDa
p-4E-BP1 S65
20 kDa
4E-BP1
35 kDa
35 kDa
p-eIF2α S51
Hypoxia - +
75 kDa
63 kDa
α-tubulin for p-eIF2α
(not used in final figure)
35 kDa
eIF2α
63 kDA
α-tubulin

Figure 2 a) Additional replicates

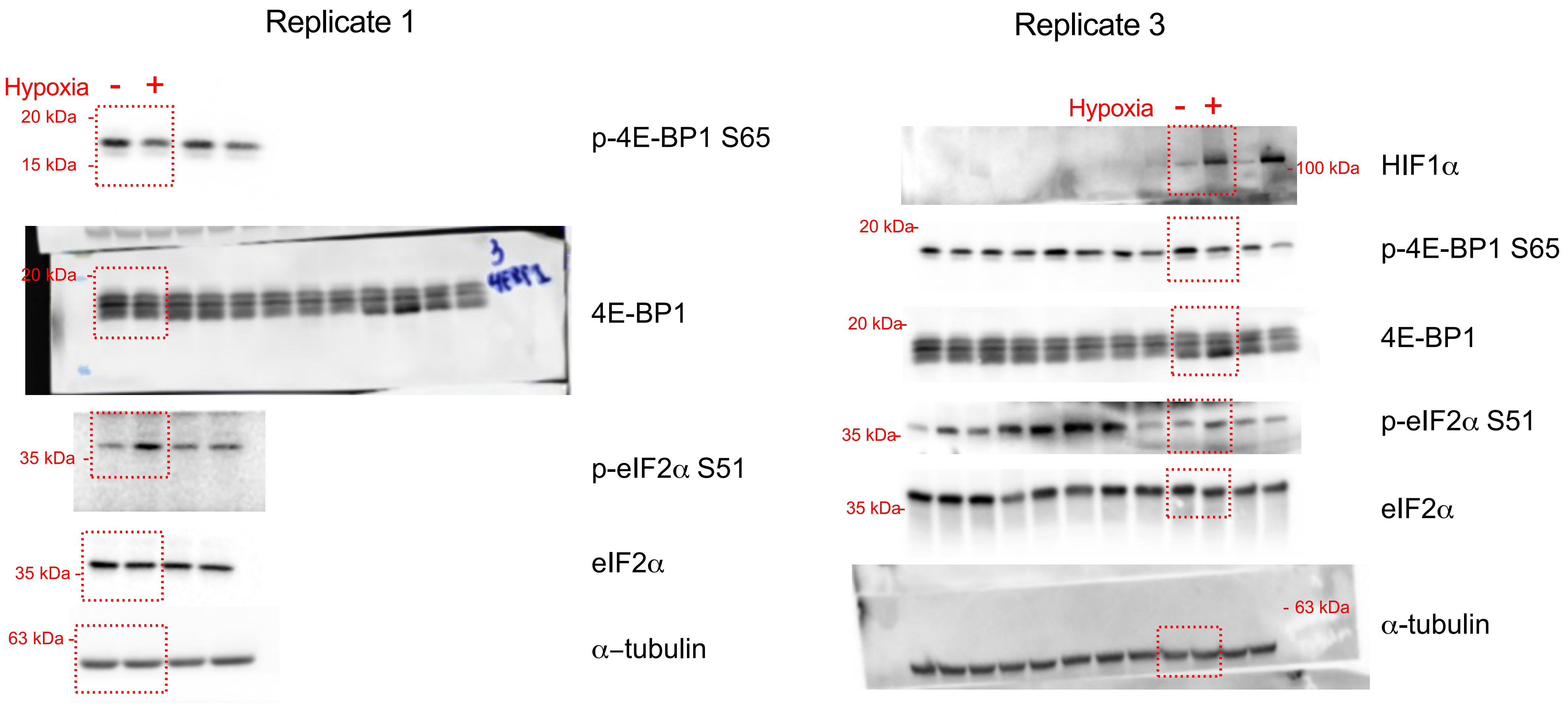

Figure 2 b)

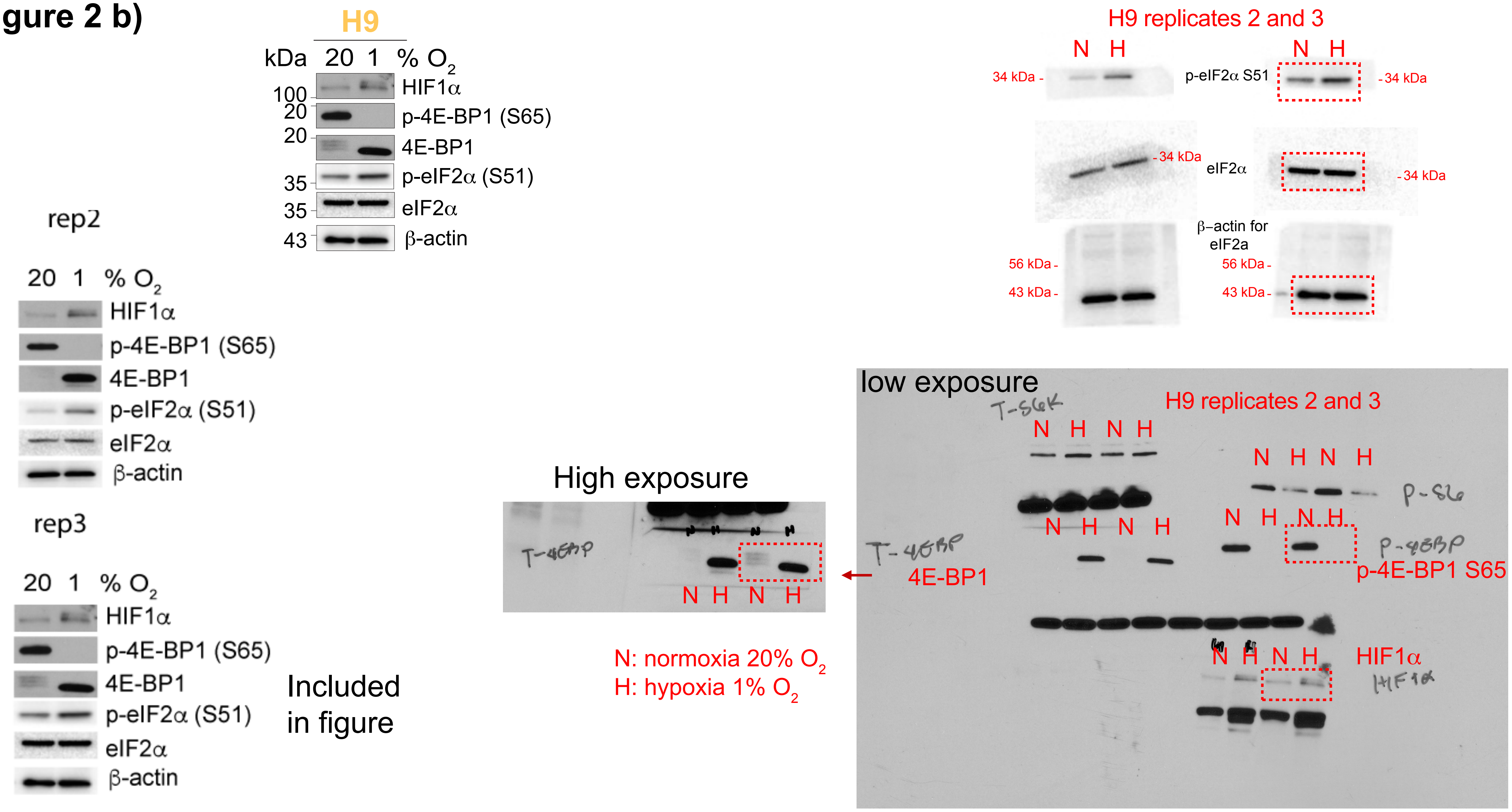
H9
kDa 20 1 % O2
100
20
20
35
35
43
HIF1α
p-4E-BP1 (S65)
4E-BP1
p-eIF2α (S51)
eIF2α
β-actin
rep2
20 1 % O2
HIF1α
p-4E-BP1 (S65)
4E-BP1
p-eIF2α (S51)
eIF2α
β-actin
rep3
20 1 % O2
HIF1α
p-4E-BP1 (S65)
4E-BP1
p-eIF2α (S51)
eIF2α
β-actin
Included in figure
H9 replicates 2 and 3
N H
N H
34 kDa
p-eIF2α S51
34 kDa
34 kDa
eIF2α
34 kDa
β-actin for eIF2a
56 kDa
43 kDa
56 kDa
43 kDa
High exposure
T-4EBP
N H N H
4E-BP1
N: normoxia 20% O2
H: hypoxia 1% O2
low exposure
T-S6K
H9 replicates 2 and 3
N H N H
N H N H
P-S6
N H N H
N H N H
T-4EBP
p-4EBP
p-4E-BP1 S65
N H N H
HIF1α
HIF1α

Figure 4 a)

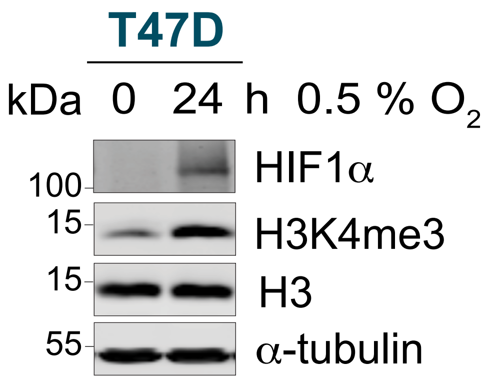

Replicate 1
included in
figure

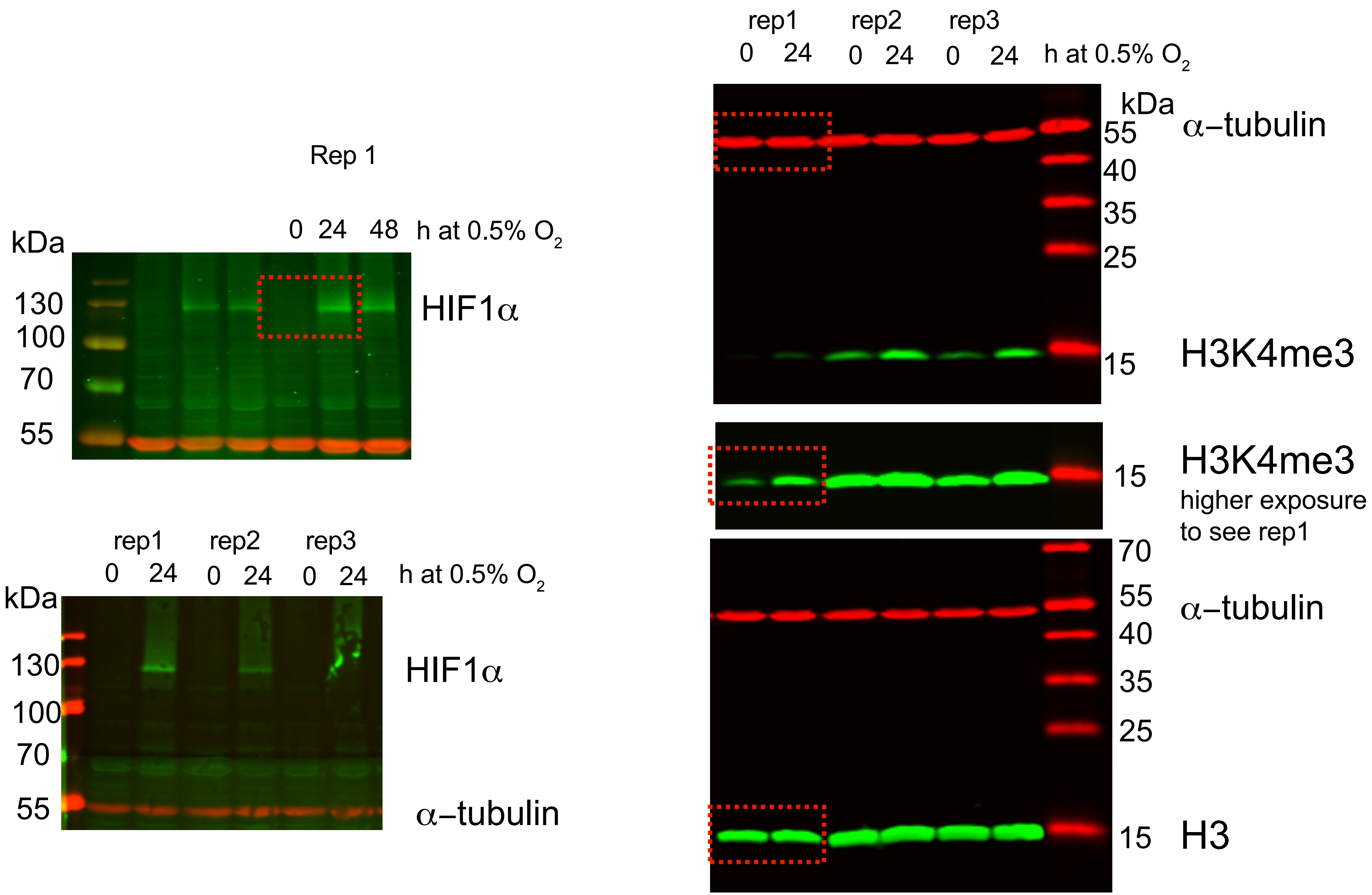

Figure 4 f)

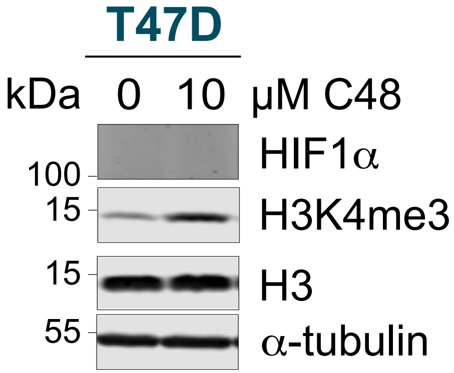

Replicate 2 included in figure

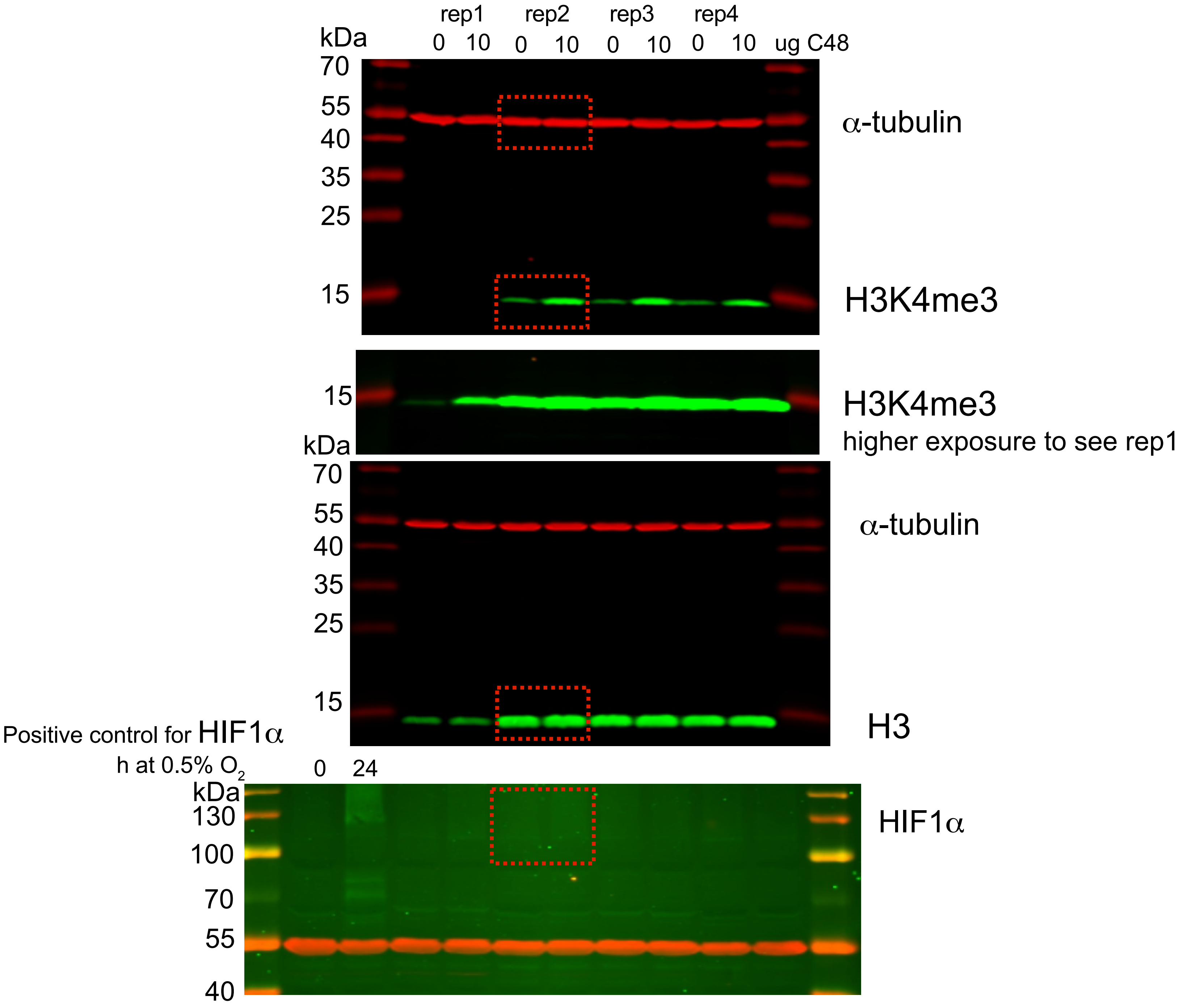

Figure 5 a)

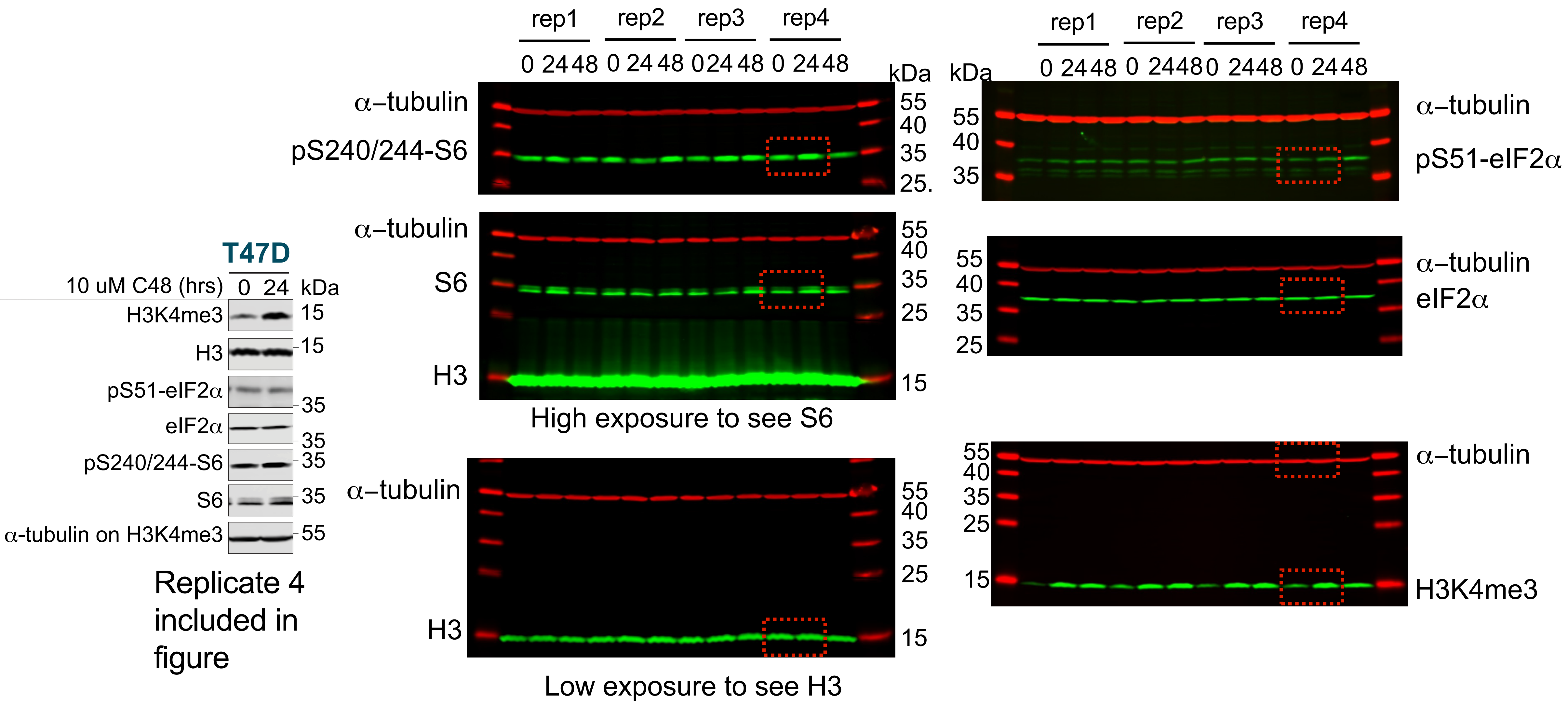

T47D
10 uM C48 (hrs)
0
24
kDa
H3K4me3
H3
pS51-eIF2α
eIF2α
pS240/244-S6
S6
α-tubulin on H3K4me3
Replicate 4 included in figure
rep1
rep2
rep3
rep4
0 24 48
α–tubulin
pS240/244-S6
S6
H3
High exposure to see S6
Low exposure to see H3
pS51-eIF2α
eIF2α
H3K4me3
55
40
35
25
15

Figure 6 a)

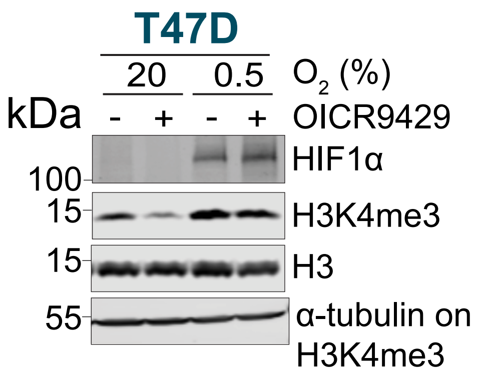

Replicate 3
included in
figure

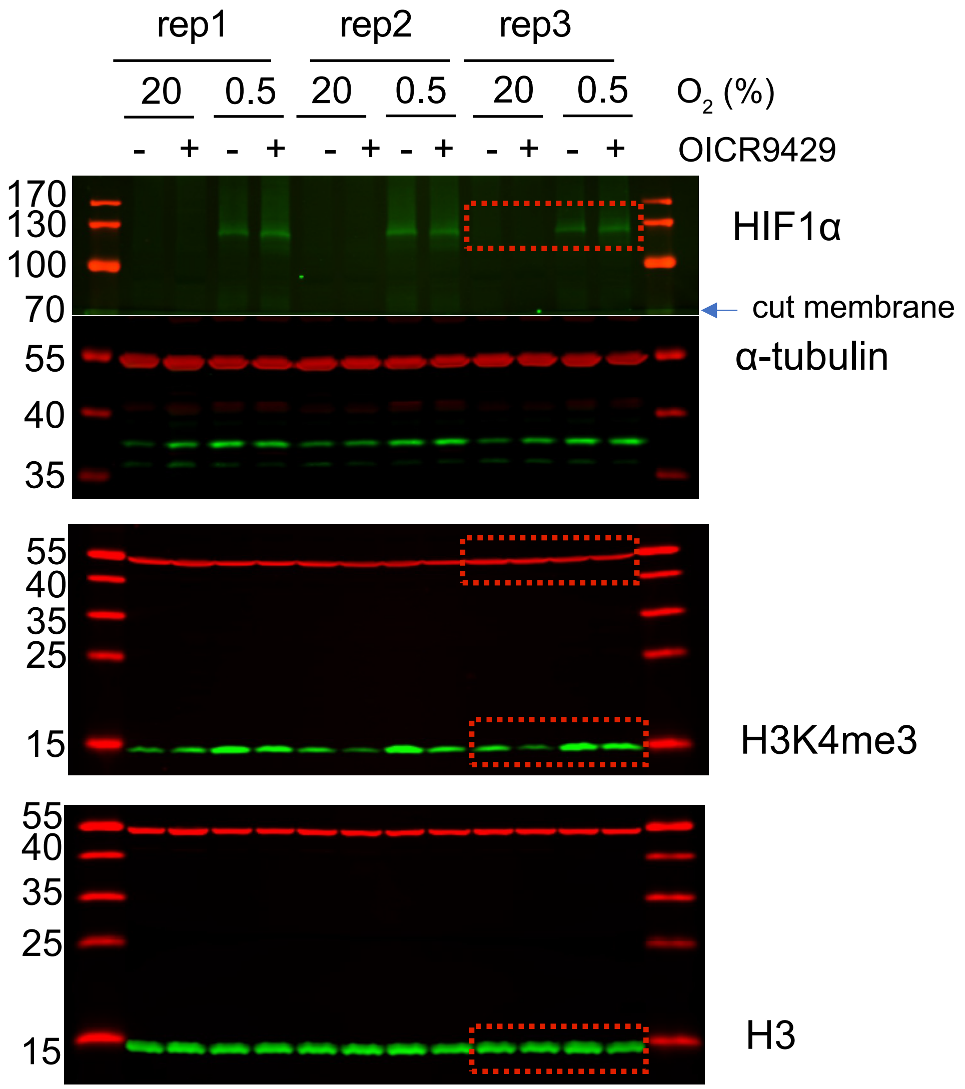

Figure 7 b)

Rep1

2021_02_25

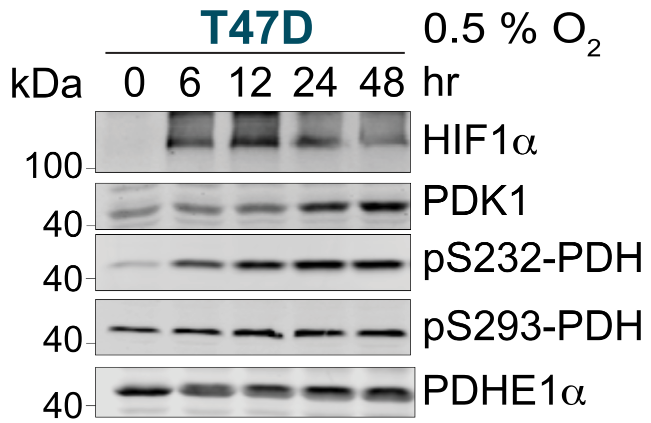

Replicate 1 included in figure

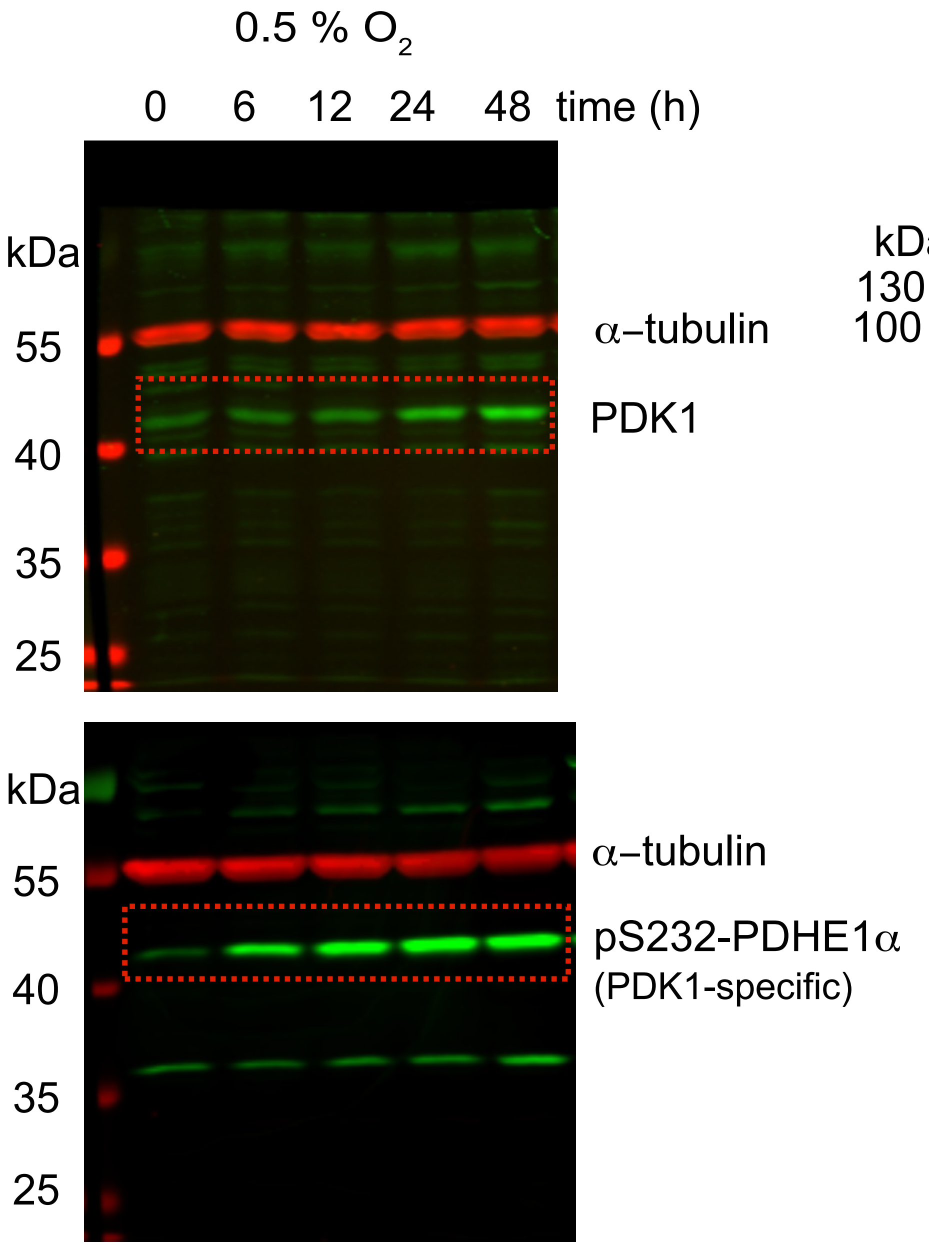

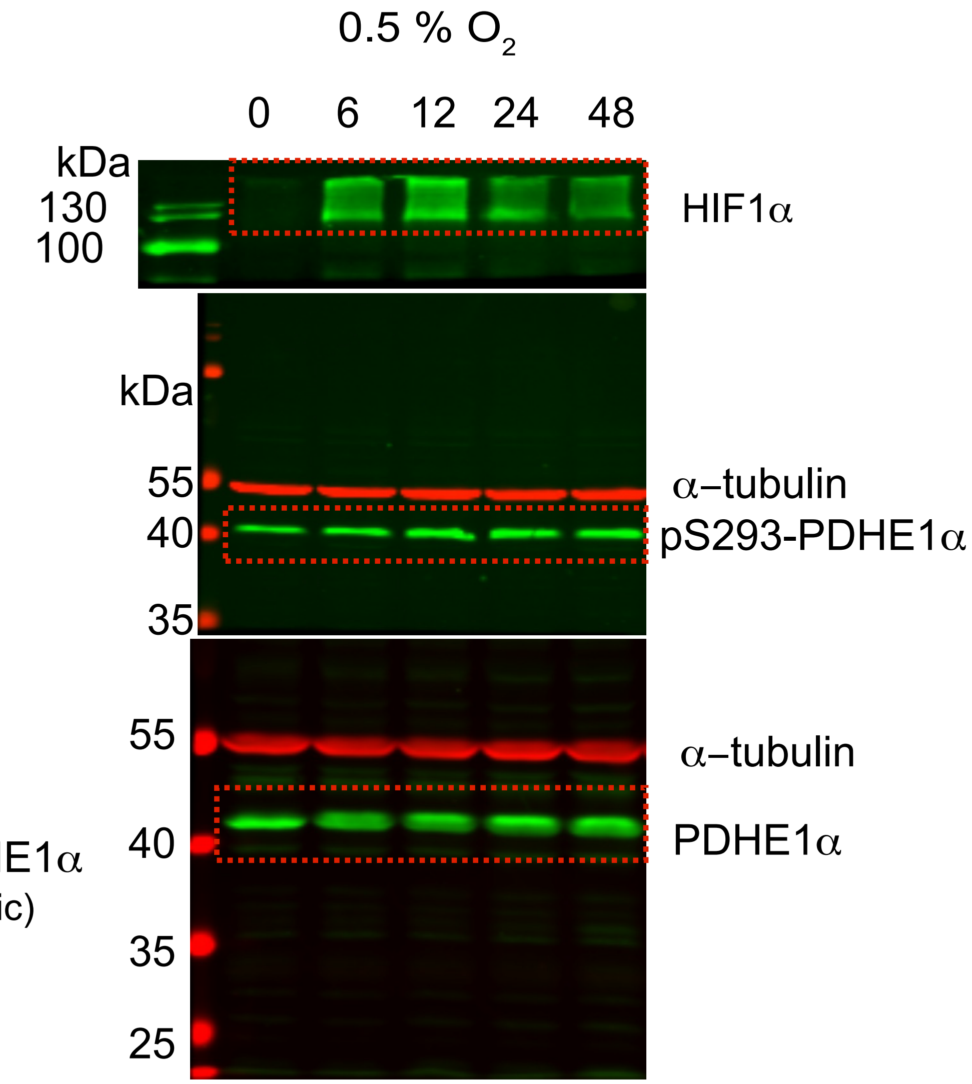

Figure 7 b) Additional replicates

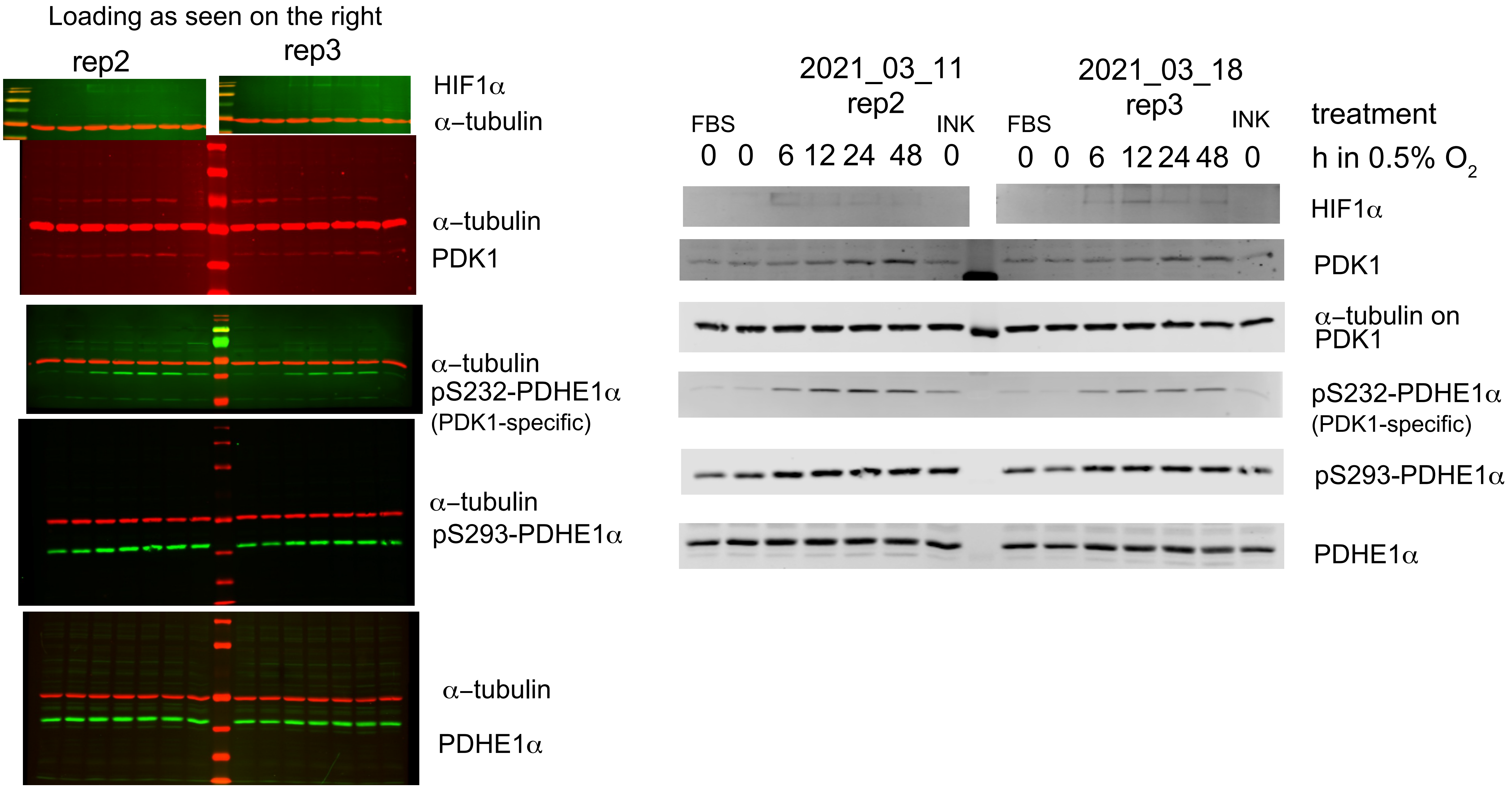

Figure 7 i)

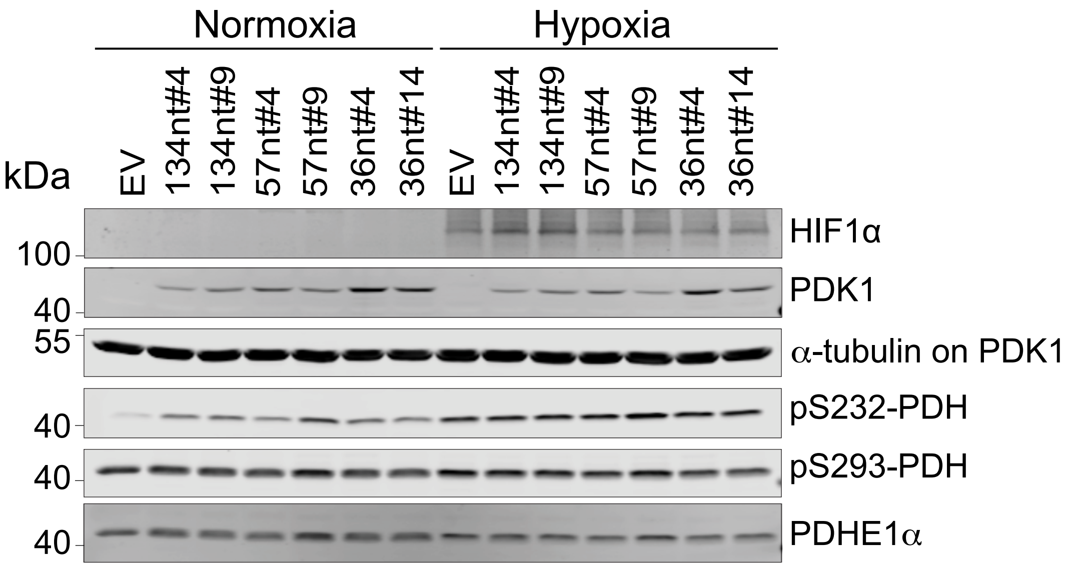

Replicate 1 included in figure

Rep1 loading as on left

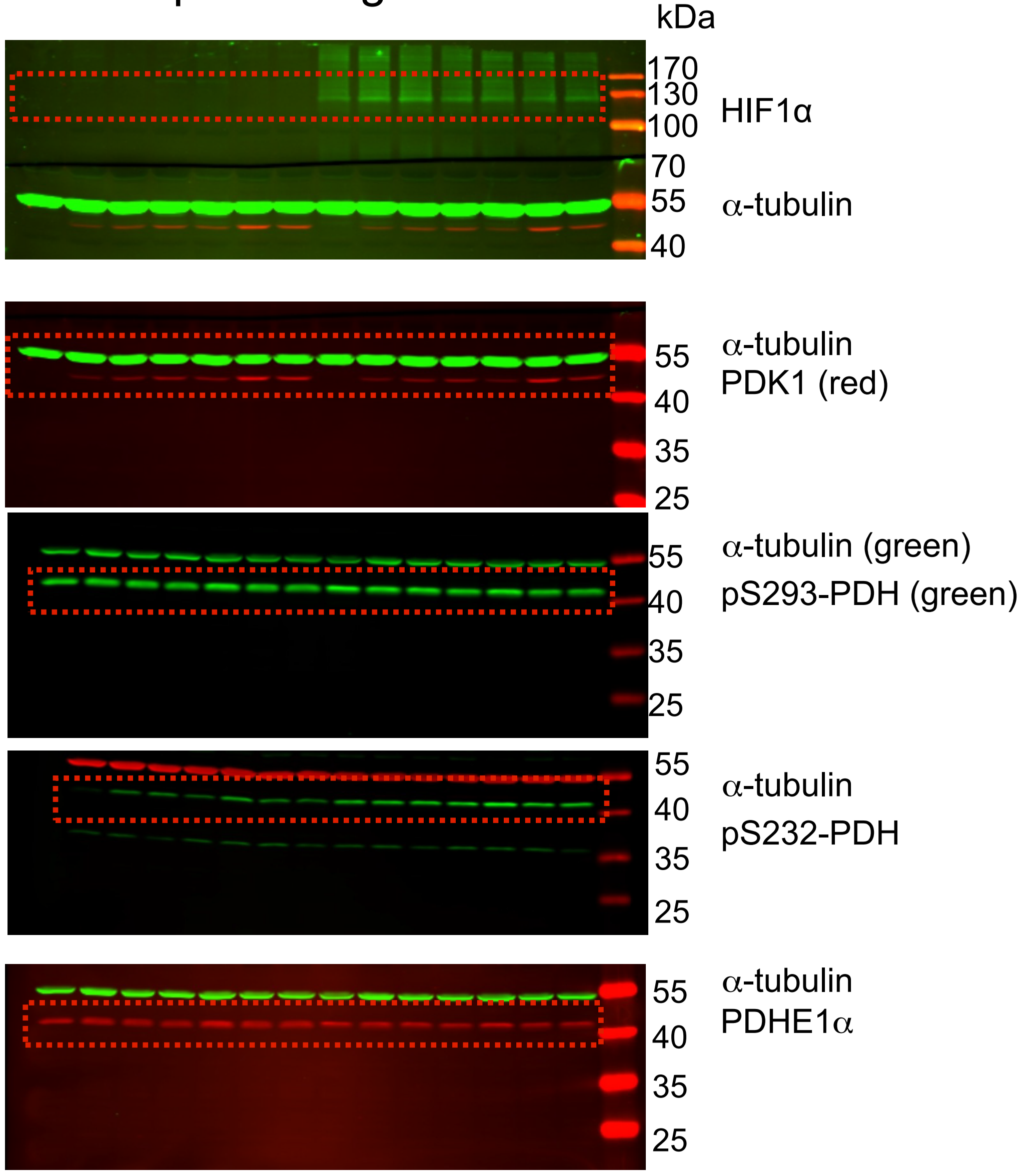

Figure 7 i) Additional replicate

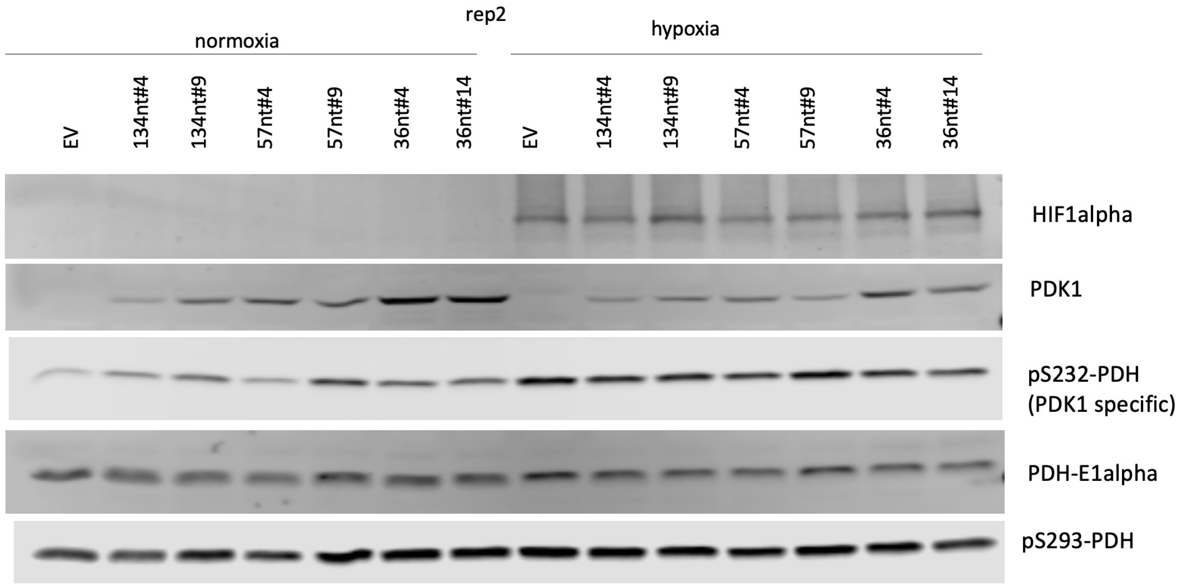

Rep2 loading as on left

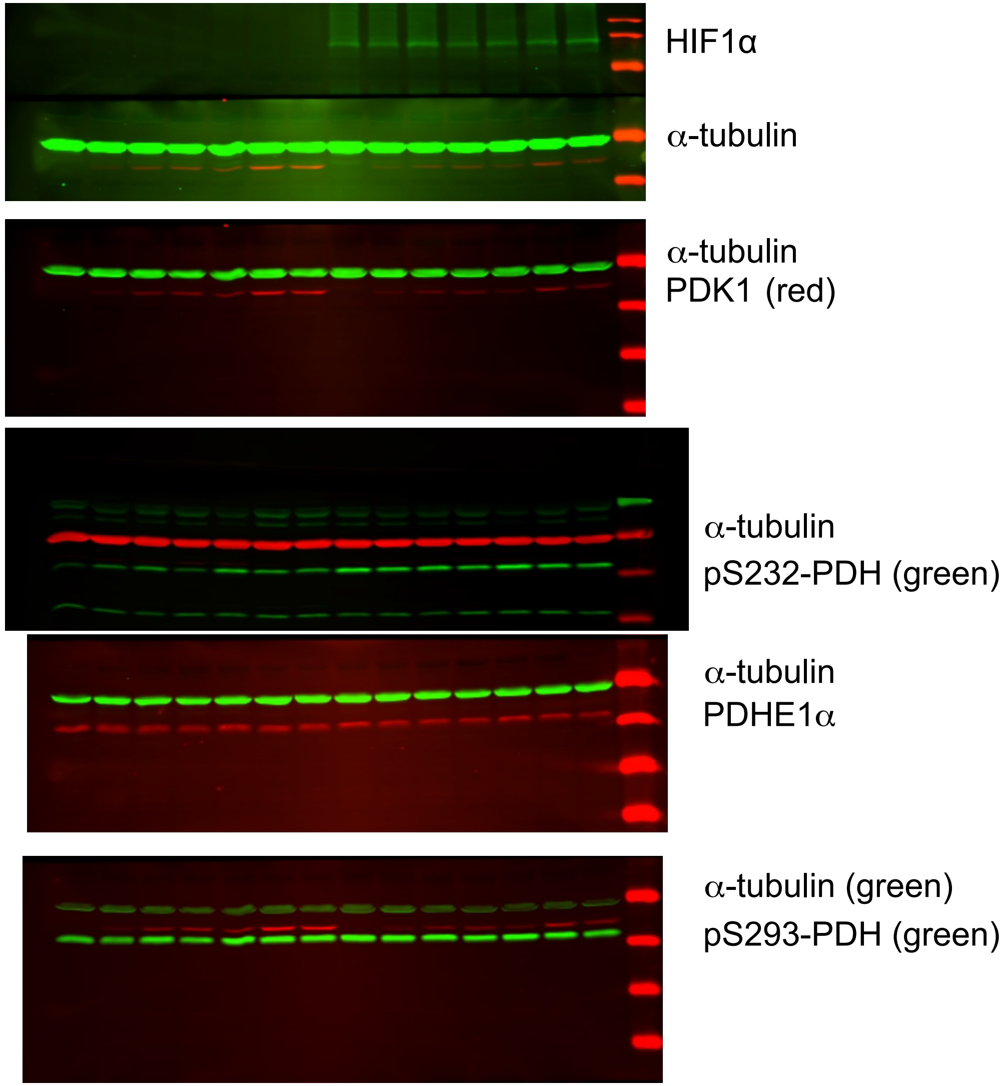

Extended Data Figure 6d)

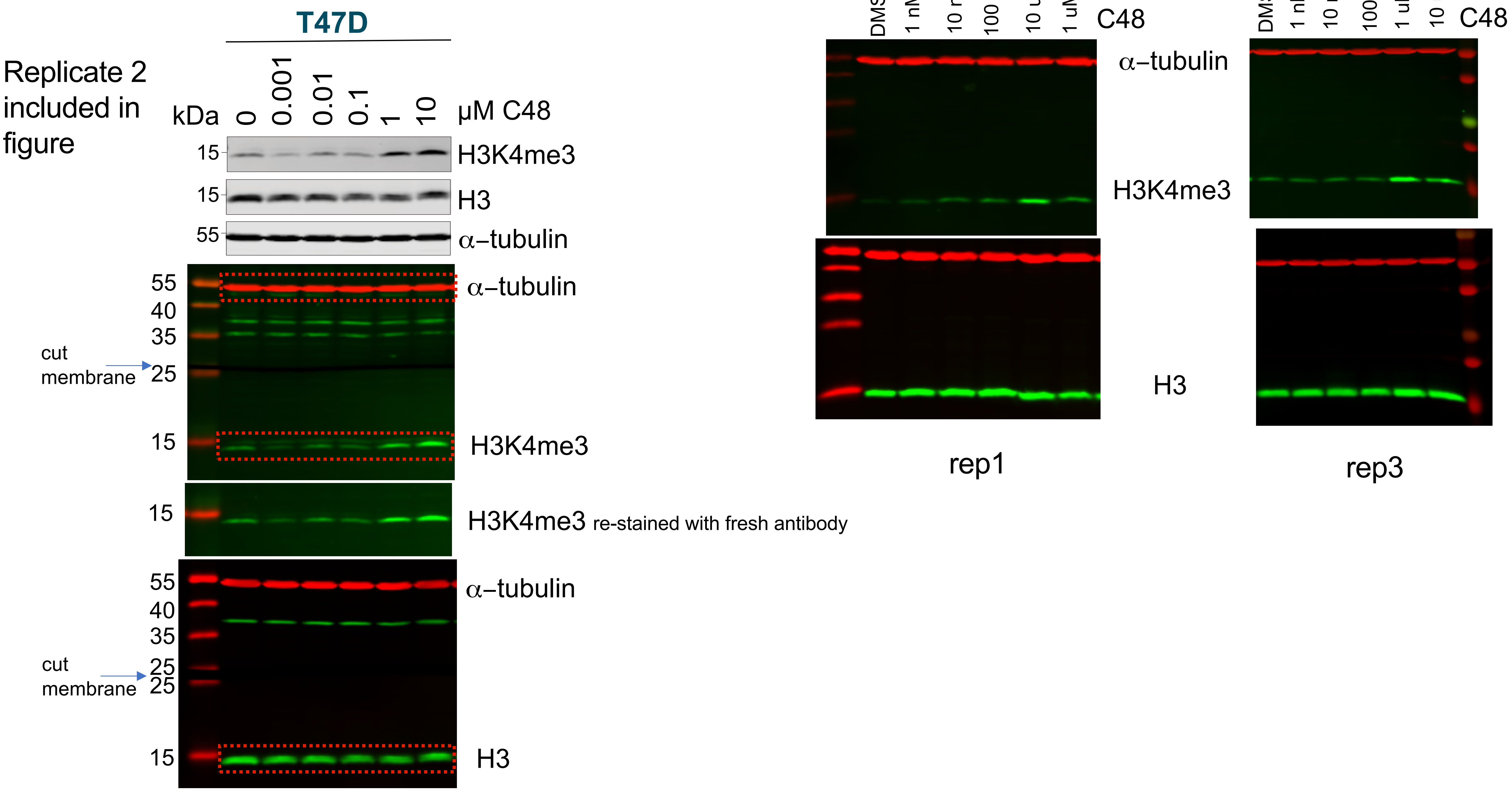

Extended Data Figure 8 e)

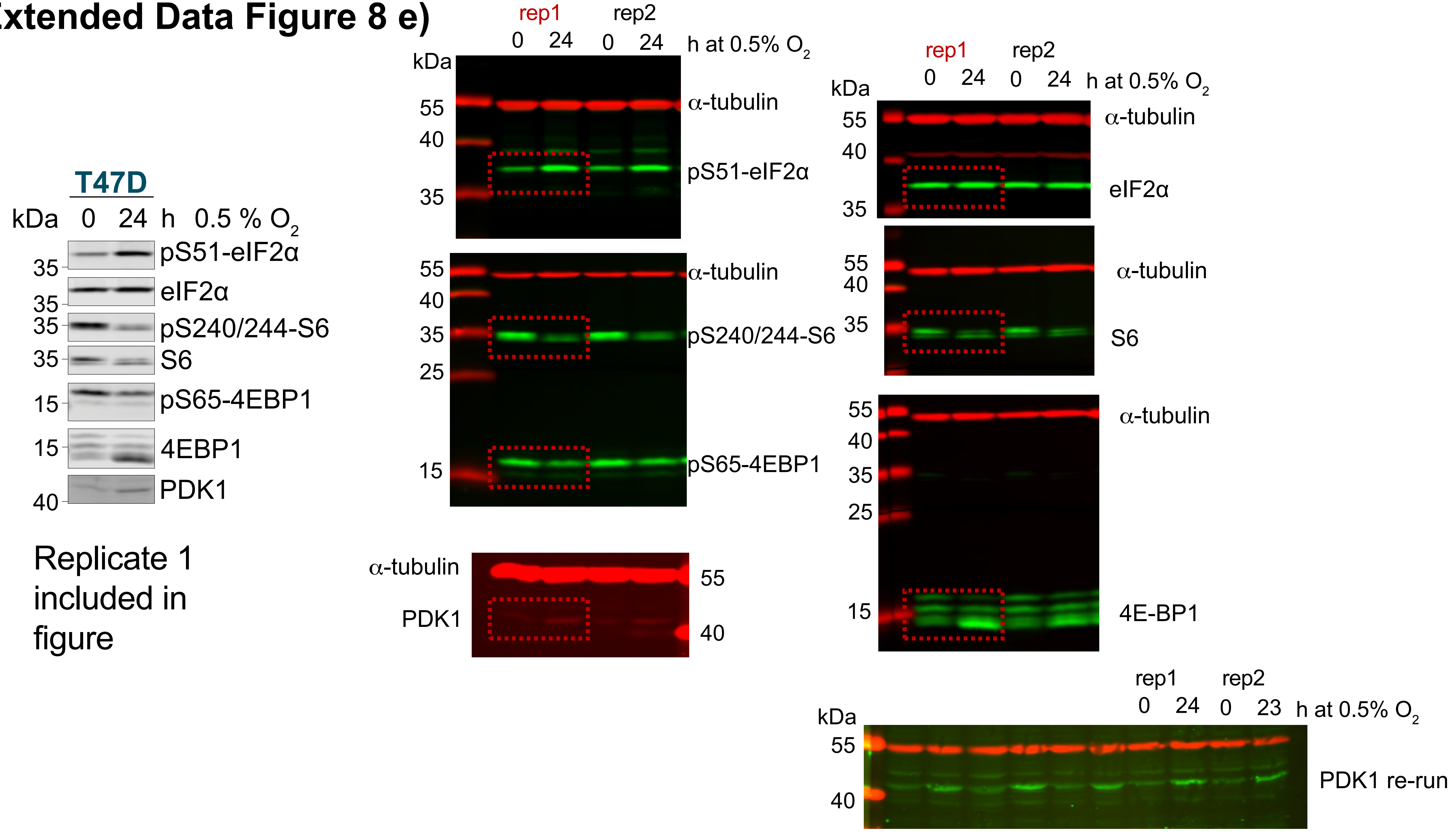

Extended Data Figure 9 a)

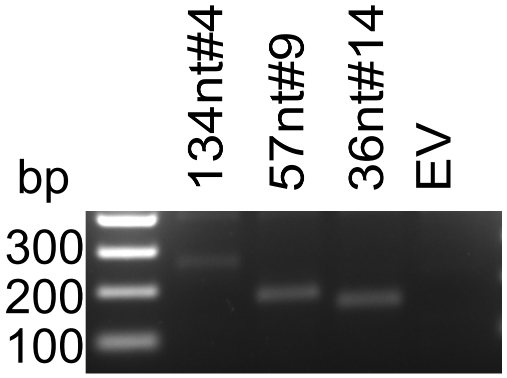

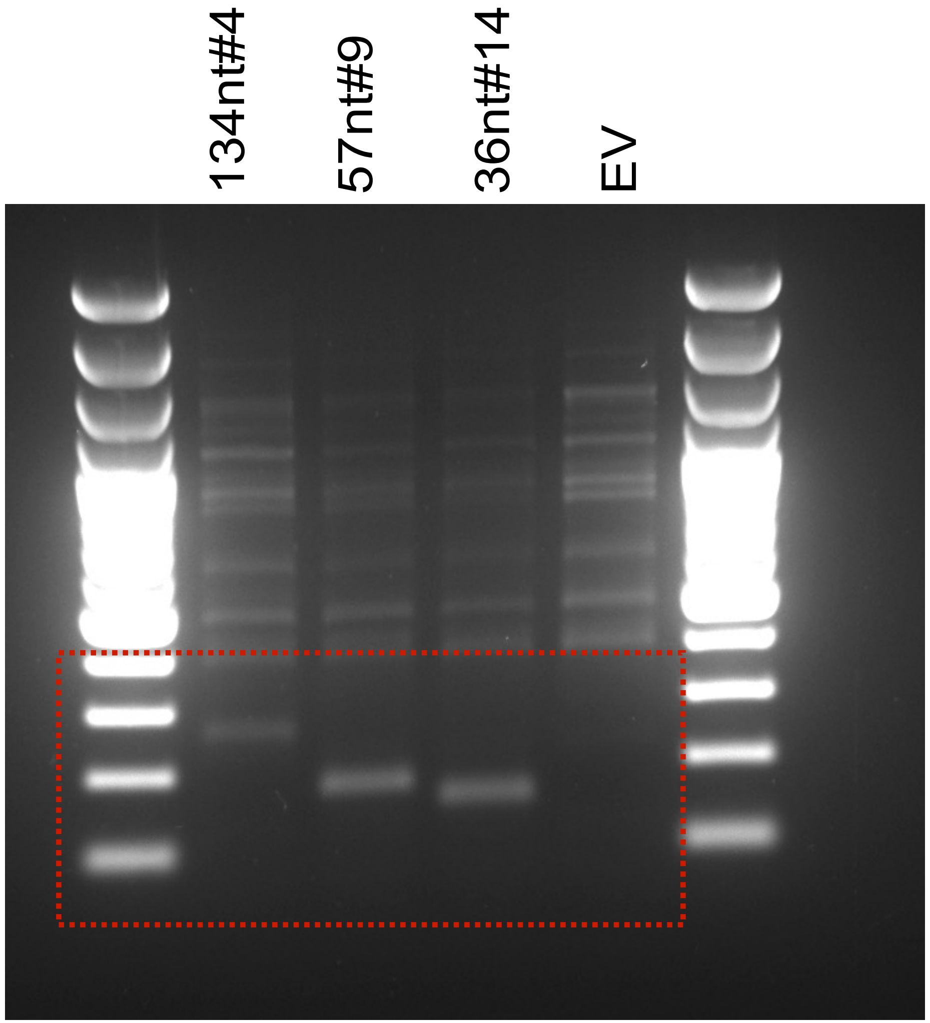

Supplement: Supplementary file 7 — Unprocessed western blots and/or gels. [file 41556_2025_1786_MOESM7_ESM.pdf]
